# Blood Transcriptome Profiling Links Immunity to Disease Severity in Myotonic Dystrophy Type 1 (DM1)

**DOI:** 10.3390/ijms23063081

**Published:** 2022-03-12

**Authors:** Sylvia Nieuwenhuis, Joanna Widomska, Paul Blom, Peter-Bram A. C. ‘t Hoen, Baziel G. M. van Engelen, Jeffrey C. Glennon, on behalf of the OPTIMISTIC Consortium

**Affiliations:** 1 Center for Molecular and Biomolecular Informatics (CMBI), Radboud Institute for Molecular Life Sciences, Radboud University Medical Centre, 6500 HB Nijmegen, The Netherlands; sylvia.nieuwenhuis@radboudumc.nl (S.N.); peter-bram.thoen@radboudumc.nl (P.-B.A.C.‘t.H.); 2 Department of Cognitive Neuroscience, Donders Institute for Brain Cognition and Behaviour, Radboud University Medical Centre, 6525 EN Nijmegen, The Netherlands; joanna.widomska@radboudumc.nl; 3 VDL Enabling Technologies Group B.V., 5651 GH Eindhoven, The Netherlands; paul.blom@vdletg.com; 4 Department of Neurology, Donders Institute for Brain Cognition and Behaviour, Radboud University Medical Centre, 6500 HB Nijmegen, The Netherlands; baziel.vanengelen@radboudumc.nl; 5 Conway Institute of Biomolecular and Biomedical Research, School of Medicine, University College Dublin, D04 V1W8 Dublin, Ireland

**Keywords:** myotonic dystrophy type 1 (DM1), RNA sequencing, blood, immunity, muscle impairment rating scale (MIRS), DM1 disease severity, pathway analysis

## Abstract

The blood transcriptome was examined in relation to disease severity in type I myotonic dystrophy (DM1) patients who participated in the Observational Prolonged Trial In DM1 to Improve QoL- Standards (OPTIMISTIC) study. This sought to (a) ascertain if transcriptome changes were associated with increasing disease severity, as measured by the muscle impairment rating scale (MIRS), and (b) establish if these changes in mRNA expression and associated biological pathways were also observed in the Dystrophia Myotonica Biomarker Discovery Initiative (DMBDI) microarray dataset in blood (with equivalent MIRS/DMPK repeat length). The changes in gene expression were compared using a number of complementary pathways, gene ontology and upstream regulator analyses, which suggested that symptom severity in DM1 was linked to transcriptomic alterations in innate and adaptive immunity associated with muscle-wasting. Future studies should explore the role of immunity in DM1 in more detail to assess its relevance to DM1.

## 1. Introduction

Myotonic dystrophy type 1 (DM1) is a multi-system disease with, among others, a variety of neuromuscular and central nervous system (CNS) features. Patients suffer from myotonia, muscle weakness, and muscular dystrophy [1]. In addition, cardiac abnormalities, such as conduction defects, result in a higher risk of sudden death [2]. DM1 patients often present with fatigue and symptoms related to failure of smooth muscle in internal organs [3,4,5]. The clinical presentation of DM1 not only involves physical disabilities but also cognitive and emotional deficits, such as avoidance, apathy, and behavioral inflexibility [6,7,8].

The degree of neuromuscular impairment can be clinically assessed using the muscle impairment rating scale (MIRS) rating scale while other rating scales such as the myotonic dystrophy health index (MDHI) and DM1-Activ assess the broader phenotype [9,10,11].

The etiology of DM1 lies in the repeat length expansion of the CTG trinucleotide in the 3′ untranslated region of the Dystrophica Myotonica Protein Kinase (*DMPK*) gene on chromosome 19q.13.3 [5,12,13,14]. The transcription of these repeat lengths from this CTG tri-nucleotide into messenger RNA GUC tri-nucleotides results in the accumulation of multiple copies of the resultant mRNA in affected tissues. This toxic build-up of excessive mRNA repeats results in the trapping of different proteins in the nucleus. DM1 can be seen as an RNA-toxicity disease, in which the nuclear accumulation of aberrant *DMPK* mRNA transcripts harboring the CTG repeat expansion, is associated with splice variation in DM1 [15].

In turn, this prevents optimal protein production and impairs cellular communication in tissues as varied as skeletal muscle, brain, and heart [16,17,18].

Currently, clinical management strategies depend on the assessment of disease severity (e.g., via the MIRS score) together with the size of the *DMPK* repeat length expansion and age at onset [19]. Due to variant repeats and other disease modifiers, patients with the same number of repeats can present with different disease severity (not necessarily more severe) [20]. Moreover, DM1 is a progressive disease which can be subtyped by time of onset and *DMPK* repeat size which is relevant to the clinical management of the different DM1 subtypes: congenital, juvenile, adult-onset, and late-onset/asymptomatic subtypes [5].

To date, a range of biomarkers in different tissue types have been suggested for DM1, including those in urine and muscle utilizing both transcriptomic and Magnetic Resonance Imaging (MRI) metrics [21,22]. Studies in urine examining mRNA splice variants in DM1 have attempted to assess symptom severity and/or identify new therapeutic targets [22]. Gene expression profiling in peripheral blood may provide an alternative image of the systemic aspects of the disease. It has been used to assess disease progression and therapeutic response in other diagnostic cohorts [23]. It was previously demonstrated that blood miRNA profiling could be used to differentiate between DM1 patients and healthy controls [24]. While these studies are important to delineate DM1 diagnostic cohorts and muscle-related symptoms, there is a need for a surrogate biomarker profile in blood which assesses disease severity. This is the primary goal of the current study which seeks to identify transcriptomic (mRNA expression) markers of DM1 severity independent of repeat length of expansion. This is relevant as DM1 severity is not directly proportional to the size of the *DMPK* repeat length expansion with some patients presenting with a high number of repeats but relatively mild phenotype [25].

Here, we explore the molecular signatures in RNA sequencing data from blood associated with disease severity as measured in DM1 patients with less than 400 CTG-repeat length size in the *DMPK* gene in blood. These DM1 patients participated in the OPTIMISTIC study [26]. This approach involved stratifying those within the OPTIMISTIC study into different patient groups with different degrees of disease severity (as measured by the muscle-impairment rating scale (MIRS)) and assessed at baseline. Patients were divided into groups with mild (MIRS 1–2) and severe (MIRS 3–5) neuromuscular symptoms with different *DMPK* repeat length characteristics. In addition, we sought to independently validate any findings in an independent cohort namely, those with equivalent MIRS/*DMPK* repeat length data from the Dystrophia Myotonica Biomarker Discovery Initiative (DMBDI) mRNA microarray datasets in blood [27].

## 2. Results

### 2.1. Descriptive Analyses of the OPTIMISTIC and DMBDI Samples

Characteristics of the patients included in the study are described in Table 1 including age of onset, their modal CTG repeat lengths, and MIRS scores. Across the three groups, as expected, earlier onset of symptoms was associated with greater Modal CTG repeat size, as well as disease severity assessed by MIRS. The inclusion- and exclusion criteria are specified in Figure S1.

### 2.2. Data Analysis of the Transcriptome of DM1 Patients Cohorts

#### RNA Sequencing and Exploratory Analyses in OPTIMISTIC Cohort

We used RNA sequencing to measure whole-blood gene expression profiles in 30 DM1 patients. Following data filtering and normalization, we quantified 18,034 genes as being robustly expressed in our dataset, out of which 17,846 were successfully annotated with IPA. Principal Component Analysis on the normalized gene expression matrix identified two distinct clusters in the data separated based on scores on the first principal component (Figure S2.1A–D in Supplementary Materials). Different coloring schemes applied to investigate if the separation was associated with a specific factor revealed that the two clusters correspond to sex (Figure S2.1A), suggesting an overall differential gene expression between the two sexes. Sex differences in DM1 have been described elsewhere [28]. To account for this effect, sex was added to the linear model in detection of differentially expressed genes.

### 2.3. Differential Gene Expression

**Figure 1:**
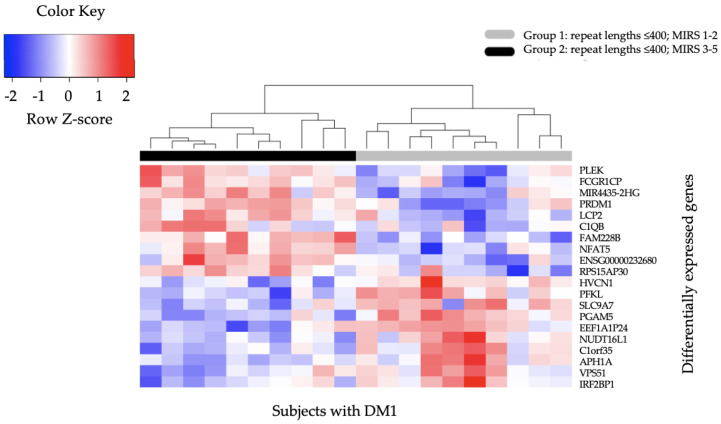
Hierarchical Clustering Heatmap of top 20 differentially expressed genes in DM1 OPTIMISTIC Group 1 and Group 2 patients. The heatmap presents the top 20 significant differentially expressed genes in subjects with DM1 from Group 2 (CTG repeat length ≤ 400, MIRS score 3–5) compared to Group 1 (CTG repeat length ≤ 400, MIRS score 1–2). Columns represent subjects with DM1, while rows represent specific genes of interest. The Z-score presents a measure of distance, in standard deviations, away from the mean. The color indicates the differentially expressed genes, with red indicating 10 higher expressed genes in Group 2 compared to Group 1 and with blue 10 lower expressed genes.

We found that 683 genes were differentially expressed in the OPTIMISTIC Group 2 (CTG repeat length ≤ 400, MIRS score 3–5) compared to Group 1 (CTG repeat length ≤ 400, MIRS score 1–2) at *p*-value ≤ 0.01. A full list of the 683 differentially expressed genes is visualized using a hierarchical clustering heatmap in Figure S2.2 in Supplementary Materials (created by Script S6.5) with the raw data shown in Table S8.1 in the Supplementary Materials. A heatmap of the top 20 genes (ranked on *p*-value), 10 with higher and 10 with lower expression in Group 2 compared to Group 1 (*PLEK*, *FCGR1CP*, *MIR4435-2HG*, *PRDM1*, *LCP2*, *C1QB*, *FAM228B*, *NFAT5*, *ENSG00000232680VPS51*, *RPS15AP30*, *HVCN1*, *PFKL*, *SLC9A7*, *PGAM5*, *EEF1A1P24*, *NUDT16L1*, *C1orf35*, *APH1A*, *VPS51,* and *IRF2BP1*) is presented in Figure 1.

**Figure 2:**
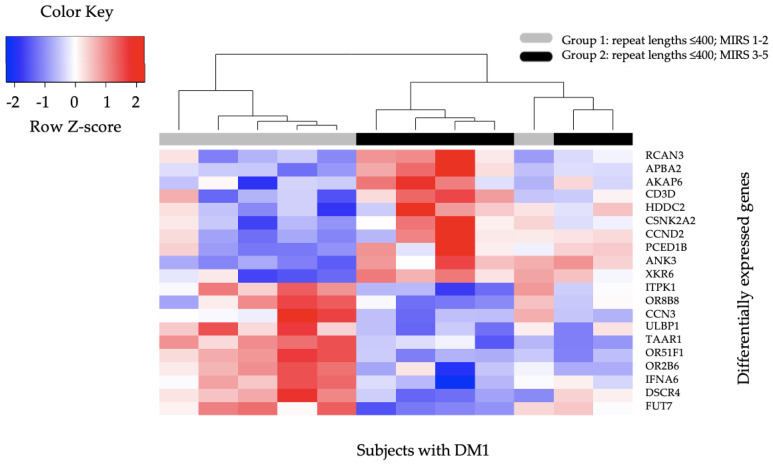
Hierarchical Clustering Heatmap of top 20 differentially expressed genes in DM1 DMBDI Group 1 and Group 2 patients. The heatmap presents the top 20 significant differentially expressed genes in subjects with DM1 from Group 2 (CTG repeat length ≤ 400, MIRS score 3–5) compared to Group 1 (CTG repeat length ≤ 400, MIRS score 1–2). Columns represent subjects with DM1, while rows represent specific genes of interest. The Z-score presents a measure of distance, in standard deviations, away from the mean. The color indicates the differentially expressed genes, with red indicating 10 higher expressed genes in Group 2 compared to Group 1 and with blue 10 lower expressed genes.

In the DMBDI dataset, we identified 225 genes that were differentially expressed genes in Group 2 (CTG repeat length ≤ 400, MIRS score 3–5) compared to Group 1 (CTG repeat length ≤ 400, MIRS score 1–2) at *p*-value (≤0.01). These 225 genes were also visualized using a hierarchical clustering heatmap approach. The selection of in total 20 genes (*RCAN3*, *APBA2*, *AKAP6*, *CD3D*, *HDDC2*, *CSNK2A2*, *CCND2*, *PCED1B*, *ANK3*, *XKR6*, *ITPK1*, *OR8B8*, *CCN3*, *ULBP1*, *TAAR1*, *OR51F1*, *OR2B6*, *IFNA6*, *DSCR4*, and *FUT7*) with the top 10 higher expressed genes and top 10 lower expressed genes in a separate heatmap shown in Figure 2. These 20 genes were ranked according to the lowest *p*-value (≤0.01).

The *p*-value distribution resulting from the test for differential expression were analysed to ensure that the difference between the groups was larger than would be expected by chance. The *p*-values for changes between Group 2 and Group 1 in the OPTIMISTIC dataset were enriched for low *p*-values (Supplementary Materials Figure S2.3) indicating that these groups indeed differ in their blood gene expression profiles. Group 3 (CTG repeat length > 400, MIRS 3–5) versus Group 2 in the same dataset, however, did not display a similar enrichment in low *p*-value distribution (Supplementary Materials Figure S2.5), suggesting that the groups with similar severity but distinct CTG repeat lengths cannot be differentiated based on blood gene expression. In the remainder of this paper we focus, therefore, on the comparison of disease severity in the Group 2 vs. Group 1 cohorts. A similar *p*-value distribution network was created for the DMBDI data (Supplementary Materials Figure S2.4), confirming a difference between the groups with different severity, albeit less strong than in the OPTIMISTC dataset.

### 2.4. Enrichment Analysis

**Figure 3:**
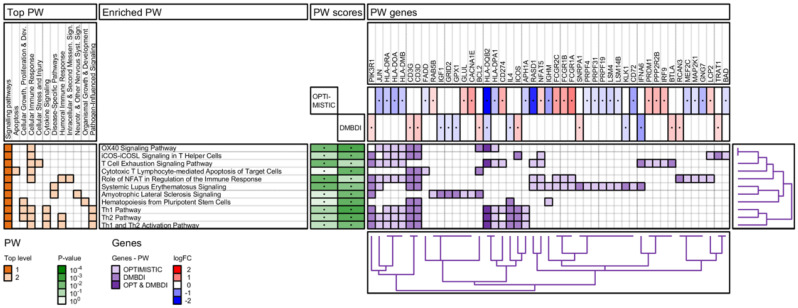
IPA pathway (PW) enrichment analysis of OPTIMISTIC and DMBDI genes. For explanation see Section 2.4.

**Figure 4:**
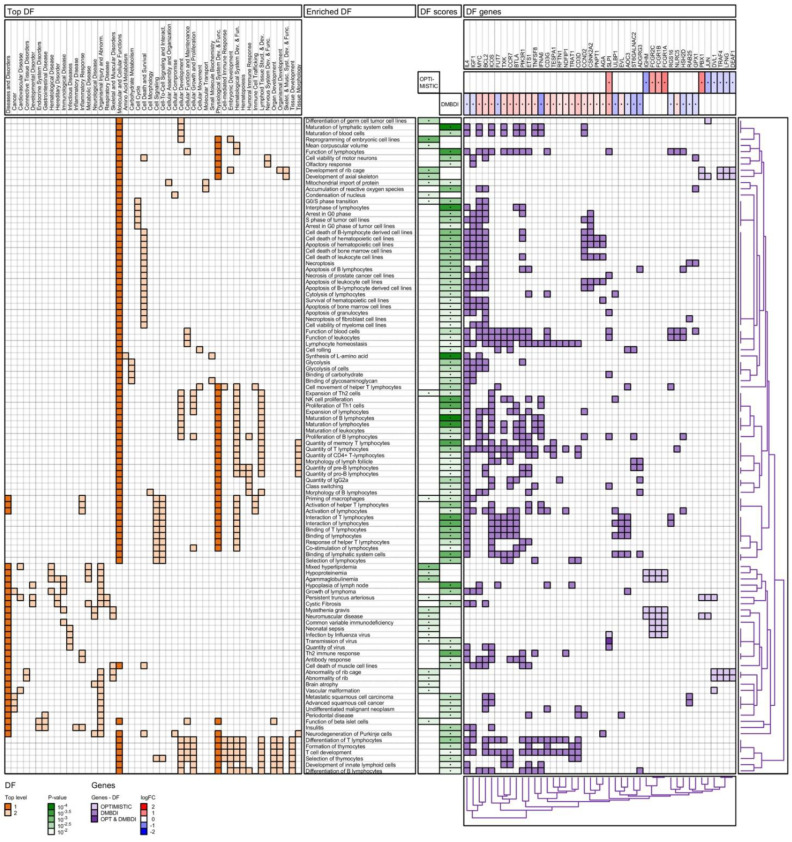
IPA diseases and functions (DF) enrichment analysis of OPTIMISTIC and DMBDI genes. For explanation see Section 2.4.

**Figure 5:**
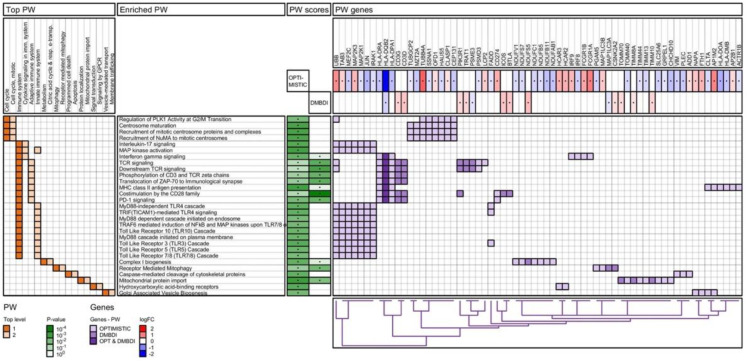
Reactome pathway (PW) enrichment analysis of OPTIMISTIC and DMBDI genes. For explanation see Section 2.4.

**Figure 6:**
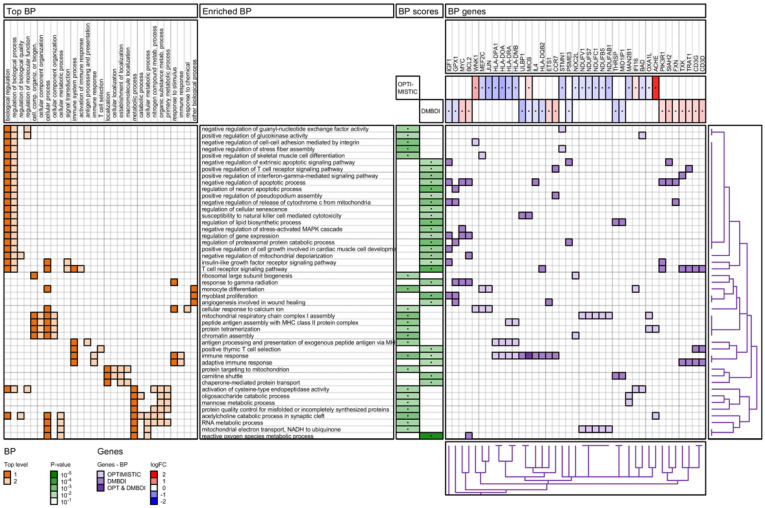
GO biological process (BP) enrichment analysis of OPTIMISTIC and DMBDI genes. For explanation see Section 2.4.

The results of the enrichment analyses are summarized in four figures (Figure 3, Figure 4, Figure 5 and Figure 6): IPA pathways, IPA diseases and functions, Reactome pathways, and Gene Ontology (GO) biological processes. For each enrichment analysis, we present gene sets which are significant (*p*-value < 0.01) for at least one (OPTIMISTIC and/or DMBDI) data set.

The heatmap in the left section shows the top-level 1 and 2 ancestor gene sets for each of the enriched gene sets. For both IPA diseases and functions (Figure 4) and GO Biological processes (Figure 6), the number of top-level ancestors is large. We therefore have removed top-level ancestors, which are less populated, for these data sets.

The middle section shows the enriched gene sets and the corresponding scores (*p*-values). The gene heatmap in the right section shows the genes present in each of the enriched gene sets. The purple color gradients show whether the genes are present in the OPTIMISTIC data set (light purple), DMBDI data set (medium purple), or in both data sets (dark purple). For each gene the corresponding log-fold change is shown for both the OPTIMISTIC and DMBDI data sets. A red–blue color gradient is used to depict the value of the log-fold change. A gene with low log-fold change will result in a (almost) white color, which makes it difficult to distinguish from a gene which is not in the gene set. Therefore, the genes present in the gene sets are marked with a black dot. The same technique is used for the *p*-values of the enriched gene sets.

Hierarchical clustering is used to organize the genes, based on the genes heatmap. The corresponding dendrogram is shown on the bottom of genes heatmap. Hierarchical clustering is also used to group and/or cluster top-level 1 and 2 ancestor gene sets based on the heatmap in the left section (except for the Reactome pathways). The corresponding dendrogram is shown on the right of genes heatmap.

#### 2.4.1. IPA Pathway Analysis

No overlap in differentially expressed genes is found between the OPTIMISTIC and DMBDI datasets. However, we found converging mechanisms on the IPA gene set enrichment level. Within the OPTIMISTIC dataset, we found four pathways which were significantly enriched (OX40 Signaling Pathway; T-cell Exhaustion Signaling Pathway; Role of NFAT in Regulation of the Immune Response Pathway; and Systemic Lupus Erythematosus (SLE) Pathway), while in the DMBDI dataset, these four pathways plus a number of additional pathways were significantly enriched, particularly those involving immune function (Figure 3). Of note, two enriched disease specific pathways, SLE and amyotrophic lateral sclerosis (ALS) share differentially expressed genes with many immune-related pathways. The enriched OX40 Signaling Pathway was significantly enriched in both the OPTIMISTIC and DMBDI datasets, which is part of the top-level pathway ‘Cellular Immune Response’. Within this top-level pathway category, a number of DMBDI enriched pathways are seen, notably iCOS-iCOSL Signaling in T Helper Cells, T-cell Exhaustion Signaling Pathway, Cytotoxic T Lymphocyte-mediated Apoptosis of Target-cells, Role of NFAT in Regulation of the Immune Response Pathway, and Th1/Th2 (activation) pathways (Figure 3). Furthermore, in both the top-level pathways, Cytokine Signaling and Pathogen influenced signaling, a role for enriched Th1/Th2 (Activation) pathways was highlighted. The top-level pathway Humoral immune response implicates a number of enriched pathways, including the ‘Role of NFAT in Regulation of the Immune Response Pathway’, the Th2 pathway, and Th1 and Th2 activation pathways.

#### 2.4.2. IPA Diseases and Functions Analysis

We observed enrichment in both the OPTIMISTIC and DMBDI datasets within three main top-level 1 categories as shown in Figure 4 (dark orange colors). The first category, ‘Diseases and Disorders’, shows immune-related, metabolic, neurological, skeletal, and muscular (e.g., agammaglobulinemia, vascular malformation, Th2 immune response, insulitis, Myasthenia gravis, and neuromuscular disorder) pathways. The second and third main top-level 1 categories show some overlap in Molecular and cellular functions together with Physiological System Development and function, particularly in a range of processes affecting B and T lymphocytes, notably their life cycle, function, activity, and homeostasis pathways. There are some shared biological functions between the OPTIMISTIC and DMBDI datasets, such as mitochondrial import of protein, priming of macrophages, and expansion of Th2 cells.

#### 2.4.3. Reactome Pathway Analysis

As shown in Figure 5, the Reactome pathways analyzed can be divided in two main top-level 1 categories; Cell cycle and Immune system. Regulation of the cell cycle is a prominent feature in the DMBDI dataset but is not shared with the OPTIMISTIC dataset. In contrast, the main overlap between both the OPTIMISTIC and DMBDI datasets is pathways involving adaptive immunity, consistent with the IPA analysis. Within, the immune system top-level 1 category, there is enrichment in three pathways. The first of these involves Cytokine signaling in immune system (e.g., IL17-, IFNγ signaling, and MAP kinase activation). The second involves Adaptive immunity (e.g., T-cell receptor (TCR) related signaling and major histocompatibility complex class II (MHCII) antigen presentation). The third enriched pathway is innate immunity (e.g., MyD88 related signaling and Toll-like receptor (TLR) related cascades).

#### 2.4.4. Gene Ontology (GO) Biological Process Analysis

The results of the GO biological process analysis are shown in Figure 6. Of these, the top-level 1 categories highlight a role for cellular processes, particularly those involving the immune, metabolic and stimulus–response aspects. Interestingly, the GO biological processes also implicate a role for the cellular immune response and highlight enriched biological processes where T-cells play an important role. The cellular immune response is also the only shared biological process between the OPTIMISTIC and DMBDI datasets. In addition, enriched pathways implicating apoptosis are also highlighted.

### 2.5. Master Regulators of Gene Expression

**Figure 7:**
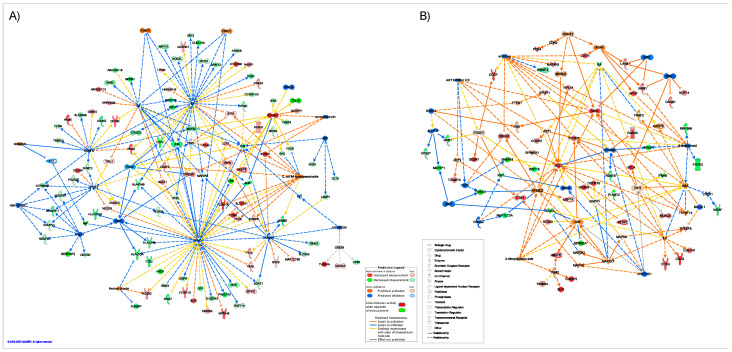
Causal Network analysis of interacting master upstream regulators based on differentially expressed genes in the OPTIMISTIC and DMBDI datasets. Causal networks of “significant” master regulators and their target genes are shown for (**A**) OPTIMISTIC and (**B**) DMBDI datasets.

Causal Network analysis identified interacting master upstream regulators based on genes that were differentially expressed in OPTIMISTIC and DMBDI datasets. In Figure 7, we present networks of “significant” master regulators and their target genes in OPTIMISTIC (Figure 7A) and DMBDI (Figure 7B) datasets. The full list of master regulators for OPTIMISTIC and DMBDI is presented in the Supplementary Materials (Tables S9.1 and S10.1, respectively). In the OPTIMISTIC dataset, we identified 17 master regulators. The top ranked transcription factors FOXD1 and FOXJ1, indirectly (through other regulators: IFNγ, IL4, and IL2) target 71 differentially expressed genes and are predicted to be activated. Three other transcription regulators (PAX5, CIITA, and SALL4) are predicted to be inhibited and interact with other regulators in the network. Other regulators include growth factor VEGFA, enzyme KRAS, and drugs: picropodophyllin and ADI PEG20—all predicted to be inhibited, as well as titanium dioxide and *E. coli* B4 lipopolysaccharide (LPS)—predicted to be activated. In DMBDI, transcription regulators MXI1, MLX, and MXD1 are predicted to be inhibited, while CCNT1 to be activated. Other regulators predicted to be activated include: Cytokines IL3 and TNFSF13, transporter BAX, MAP2K7 kinase, and chemical toxicant 3-nitropropionic acid, while chemical drugs/reagents: 5-fluorourcil, sirolimus, and AKT inhibitor VIII are predicted to be inhibited.

### 2.6. Splice Variant Analysis

The splice variant analysis is presented in Supplementary Materials Figures S2.6 and S2.7 does not show any significant splicing differences between the Group 2 and 1 in the OPTIMSTIC study.

## 3. Discussion

### 3.1. Limitations of the Current Study

The findings of the current study have to be seen in light of limitations, such as the small number of patient samples in each condition (which are 10 per condition) (first group; a CTG repeat length smaller than 400 with a MIRS of 1 or 2; second group CTG repeat length smaller than 400 with a MIRS of 3 to 5; and the third group CTG repeat length more than 400 with a MIRS of 3 to 5). As such the findings should be seen as a pilot study. We do not observe any significant genes expression differences after correction for multiple testing. The *p*-value distributions shown in Supplementary Materials Figures S2.3 and S2.4 suggest that the expression profiles of the two groups are distinct. Additionally, clear effects with differential effects across gender were noted. Increased sample size will be required to resolve this. The current study does not control for Intelligence Quotient (IQ), age, or ethnic group. Budgetary constraints dictated sample size.

Furthermore, this study only examines transcriptomic profiles associated with baseline DM1 and not the effect of intervention. Nor does it assess whether there are similar proteome changes as in the transcriptome. Quality analyses examining the effect of site where the study samples were collected (Newcastle, Nijmegen, Paris, and Munich) determined no site related differences in the transcriptome. This cooperation among the sites illustrates that a multi-center study is feasible in terms of harmonization of these aspects. With the current RNA sequencing depth, we will find the majority of low to medium to high abundant genes but may miss very rare low abundantly expressed genes. We are aware of the need for stringency of the current findings and acknowledge that the analysis does not correct for the false discovery rate. This reflects the current power given the relatively small sample size of the current study. The cross comparison of the microarray DMBDI dataset is still useful to compare with the RNA-seq data from the OPTIMISTIC as there is a large overlap in the exome examined both approaches. A number of other gene expression studies have examined transcriptomic signatures principally to compare DM1 with non-DM1 populations. Mis-splicing and gene expression differences in DM1 skeletal muscle link disease status with muscle strength [29]. Others report alternative splicing events in muscle as proxies of disease severity and therapeutic response [30] but are not indicated as causal in DM1 pathology [31]. Indeed our own data suggests no changes in alternative splicing with increasing DM1 severity which may suggest any splicing events that may occur in DM1 are independent of severity. Some studies have implicated MBNL2 loss of function as a critical step in DM1 pathology [30] while others have highlighted whole, differentiation stage specific, splicing complexes that are misregulated in DM1 as shown by transcriptomic analysis in DM1 myoblasts [32]. Others have reported gene expression changes in inflammatory markers associated with DM1 glial cell lines, notably the immune mediators CXCL10, CCL5, CXCL8, TNFAIP3, and TNFRSF9 [33]. Furthermore, there have been reports of upregulated interferon-regulated genes (IRGs) and genes associated with the innate immune response when comparing DM1 versus healthy controls [34]. Some transcriptomic studies have examined the role of other RNA species beyond mRNA. Notable, four circular RNAs (circRNAs) have been implicated as differentially expressed in DM1 muscle biopsies: CDYL, HIPK3, RTN4_03, and ZNF609. These circRNAs are associated with skeletal muscle strength, and those with more severe symptoms [35]. Regulatory elements such as microRNAs (miRNA) can also influence mRNA expression. Increased miRNA levels have been implicated in muscle-wasting and weakness in DM1 patients, including miR-1, miR-133a, miR133b, and miR-206 which were compared to disease stable DM1 patients [36]. The current investigation addressed mRNA related changes and does not address the potential role of other RNA species.

While it is clear that there is little/no overlap on the specific gene expression level when comparing the OPTIMISTIC and DMBDI datasets, there are converging mechanisms on the gene set enrichment level—pathways and GO terms between the two datasets. These introduce complementary information reflecting different aspects of immune system dysregulation, particularly in adaptive immunity and to a lesser degree in innate immunity. Furthermore, there is overlap between OPTIMISTIC and DMBDI on the gene enrichment level in terms of macrophage priming, mitochondrial import of proteins and Th2-cell expansion. On a higher level, adaptive immunity processes are common to both the OPTIMISTIC and DMBDI datasets. However different aspects/pathways within adaptive immunity are highlighted in the two datasets respectively.

The design of the OPTIMISTIC study is a within-DM1 intervention study (only involving patients with different degrees of DM1 symptom severity). As such we did not have access to healthy controls to compare. Therefore, we framed the research question to investigate transcriptomic markers related to disease severity in DM1 as this is the only cohort that was recruited.

The current study sought to (a) ascertain if increasing disease severity (as measured by the muscle impairment rating scale (MIRS)) in DM1 from the EU FP7 OPTIMISTIC cohort are associated with changes in the blood transcriptome and (b) establish if these changes in mRNA expression and associated biological pathways were also observed in an independently performed DMBDI microarray dataset in blood (with equivalent MIRS/DMPK repeat length).

### 3.2. A Role for Dysregulated Immunity in DM1

**Figure 8:**
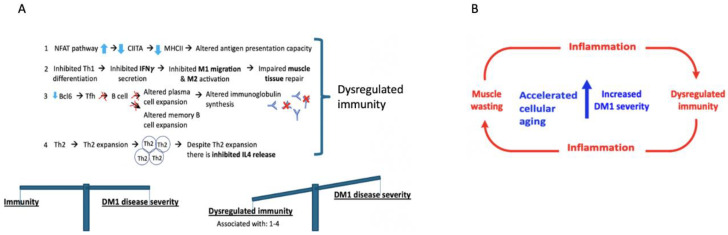
(**A**) A hypothetical framework (based on pilot data presented here) highlights four key immune processes which are possibly dysregulated. 1; higher expression of the NFAT pathway results in impaired antigen presentation capacity, through lowered *CIITA* and *MHCII* gene expression. 2; impaired Th1 differentiation which may lead to diminished IFNγ release, which normally controls macrophage subtype 1 (M1) migration and M2 activation which guide muscle tissue repair [37]. 3; diminished follicular helper T (Tfh) cell differentiation resulting in altered B memory cell expansion and altered plasma B cell expansion which may result in less antibody synthesis [38]. 4; Th2 expansion coupled with lowered *IL4* expression with increased DM1 severity. Together these affected pathways may result in dysregulated immunity resulting in a more severe DM1 phenotype. (**B**) Schematic representation of the concept that increased DM1 severity is associated with accelerated cellular aging, muscle-wasting, and dysregulation of immunity.

The current analysis suggests that DM1 severity may be associated with common biological processes namely innate and adaptive immunity. This is seen in both the OPTIMISTIC and DMBDI datasets but different aspects of innate and adaptive immunity are highlighted in the two datasets. The evidence supporting a role for adaptive immunity is observed in both IPA (Figure 3) and Reactome (Figure 5) pathway analyses where a number of processes influencing T-cell function are implicated. Reactome analyses suggest the overlap between both the OPTIMISTIC and DMBDI datasets in pathways involving adaptive immunity (e.g., T-cell receptor related signaling and antigen presentation) and cytokine signaling in the immune system (e.g., IL17-, IFNγ signaling, and MAP kinase activation). The Reactome analysis also highlights a role for innate immunity (Figure 5). Increased DM1 severity may be associated with alterations in immunity (see Figure 8A).

Adaptive immunity involves the activation of MHCII and antibody (Ab) production following the presentation of an antigen. MHCII are present on antigen-presenting cells such as phagocytes but also (skeletal) muscle cells. Both T-cell activation and expansion as well as antibody production [39,40,41,42] result from MHCII binding to an antigen. With increasing MIRS severity, a decrease in the expression of genes encoding *MHCII*, namely *HLA-DRA*, *-DOA*, and *-DMB* genes was seen in DM1. Furthermore, analysis of the master regulators of *MHCII* predicts inhibition of *IFNy* which may explain the observed down regulation of another master regulator *CIITA* in our dataset which is also a key controller of *MHCII* expression [43]. In any case, *MHCII* expression is diminished with increased DM1 severity with, as possible consequence, reduced capacity for adaptive immunity. Beyond *MHCII* capacity, adaptive immunity is involved with increasing DM1 severity as the current analyses suggest that pathways resulting in a reduced differentiation of the antibody producing plasma cells. Further evidence of a diminished adaptive immune response comes from analysis of the amount of the antibody immunoglobulin G (IgG) present in DM1 [44,45]. Total Ig (IgG, IgG1, and IgG3) levels in the blood of DM1 patients were significantly reduced compared to controls [44,45] and is associated with DM1 disease duration [46]. A hypothetical framework (which requires experimental validation) of dysregulated immunity associated with increased disease severity is shown in Figure 8B.

### 3.3. Pathway Analysis—Shared Pathways

In both the OPTIMISTIC and DMBDI studies, the data suggest pathway related changes in OX40 and SLE signaling, indicating an impairment in immunity with increasing DM1 severity [47].

OX40L–OX40 signaling is a feature of Antigen Presenting Cells (APCs) and other activated T-cells. OX40 regulates the effectiveness of antigen binding to T-cells and signals to downstream NFkB in the absence of T-cell receptor signalling. While OX40 signalling overlaps between the OPTIMISTIC and DMBDI datasets, it appears that the underlying gene expression change is in different directions. Therefore, its role in DM1 severity is difficult to interpret.

SLE signalling involves a strong immune component [48]. A connection between SLE and myotonic dystrophy was first highlighted in 1966 as both diseases are associated with muscle mass loss [49] and muscle strength impairments [50]. Clinically, SLE and DM1 also share common fatigue and daytime sleepiness symptoms. Whether the changes in immunity are associated with these common symptoms is not yet clear.

### 3.4. Pathway Analysis—Non-Shared Pathways

Some pathways are not shared by both the OPTIMSTIC and the DMBDI data set. In particular, two pathways which are only linked to the OPTIMISTIC data set, namely (i) the NFAT regulation of the immune response and (ii) the T-cell exhaustion pathway.

#### 3.4.1. NFAT Regulation of the Immune Response

NFAT is a mediator of multiple adaptive T-cell functions. NFAT modulates immune response through the transcriptional regulation of cytokines, chemokines, and growth factors in immune cells playing an essential signal for T-cell activation and proliferation. Furthermore, Ca^2+^/NFAT signaling in T-cells plays a key role in synthesizing humoral immunity, immune tolerance, and also autoimmunity (57). NFAT pathways also regulate energy and nutrient metabolism during T-cell activation by stimulation of glycolysis [51]. The NFAT pathways act to alter the expression of key immune components in Tfh cells essential for an immune response. These include a number of receptors CXCR5, ICOS, PD-1, and CD40L) on both T- and B-cells as well as cytokines (IL2, IL4, and IL21) which are necessary to synthesize catabolic Immunoglobin G (IgG) antibodies and their IgG producing plasma and memory B cells [51,52]. Decreases in IgG production may hypothetically lead to less antigen presentation resulting in an impaired immunity which is associated with higher DM1 disease severity. NFAT is also a key regulator of muscle functionality and controls activity dependent myosin switching [53] via the transcription factor *PRDM1* whose protein product BLIMP1 activates slow-twitch differentiation and represses fast-twitch differentiation [54,55]. BLIMP1 is also responsible for the transformation of CD8+ T-cells into short-lived effector cells instead of memory CD8+ T-cells. As such, its regulation by NFAT may also influence immunity in this manner.

#### 3.4.2. T-Cell Exhaustion

The second pathway which is highlighted in the OPTIMISTIC dataset is T-cell exhaustion. T-cell exhaustion is typically seen in patients with chronic viral infections that lack immunity and is the loss of effect and memory in T-cell populations. Diminished cytokine secretion in exhausted T-cells, may lead to reduced levels of the pro-inflammatory cytokines IL2 and IFNγ, possibly resulting in impaired functioning and activation of autoimmunity by continuous antigen stimulation to the T-cell receptor (TCR) [56,57]. Impaired functioning of T-cell memory leads to the lack of recall of an immune response after re-infection such that after a second viral infection, there is no synthesis of clonal antibodies to protect against infection [58,59]. Interestingly, T-cell exhaustion pathways overlap with those involved NFAT regulation of the immune response.

### 3.5. Biological Processes and Functions

Analysis of the biological processes and functions (Figure 4) again highlights immune dysregulation. A role for immune-related, metabolic, neurological, skeletal and muscular (e.g., agammaglobulinemia, vascular malformation, Th2 immune response, insulitis, Myasthenia gravis, and neuromuscular disorder) pathways across datasets is observed. In addition, processes affecting B and T lymphocytes, notably their life cycle, function, activity, and homeostasis pathways appear to be associated with increasing DM1 severity. In addition, the GO data from Figure 6 implicates a role for cellular immune responses, particularly those influencing T-cell function.

There are three shared biological processes between the OPTIMISTIC and DMBDI datasets, including (i) the mitochondrial import of protein, (ii) priming of macrophages, and (iii) expansion of Th2 cells.

#### 3.5.1. Mitochondrial Import of Proteins

The import of (precursor) mitochondrial proteins from the cytosol into mitochondria utilizes protein translocases with import signals directing each protein to the mitochondrial surface and, subsequently, to their final destination. Mitochondrial dysfunction, has been causally linked to muscle-wasting and aging [60,61]. Chronic adaptation to mitochondria-induced proteostatic stress in the cytosol may induce muscle-wasting rather than bioenergetic deficiency and oxidative stress. Beyond DM1, other muscular dystrophies, such as Facioscapulohumeral Dystrophy (FSHD) and Oculopharyngeal muscular dystrophy (OPMD), show dysregulation of mitochondrial protein import genes, such as the protein translocase ANT1 and PABPN1 (part of the TIM-23 complex), respectively [62]. Mitochondrial dysfunction in DM1 has been associated with impaired DMPK anchoring [18] resulting in oxidative metabolic impairments in DM1 and subsequent muscle-wasting [18,63,64].

#### 3.5.2. Macrophage Priming

Priming of macrophages is performed by LPS binding to TLR4 leading to a transient inflammatory response [65]. A primed macrophage is able to phagocytose the ubiquitinated proteins or antigens to the proteasome for degradation [66]. IFNγ (which is predicted to be inhibited with increased DM1 severity in Figure 8B) is a known macrophage primer cytokine capable of stimulating innate immunity. As such blunted macrophage priming leading to diminished innate immunity processes. Whether downstream ubiquitination/degradation mechanisms are also impaired with increasing DM1 severity remains to be seen. Interestingly, in SLE, an autoimmune condition (which shares aspects of the DM1 phenotype), upregulation of proteasomes is also seen [67,68,69].

#### 3.5.3. Th2 Cell Expansion

Th2 development and expansion are triggered by IL4-producing cells, such as activated T-cells and under the control of the calcineurin/NFAT-dependent pathway [70] and activation of the ICOS signaling pathway. By regulating Th2 cell number, it can impact on autoimmunity. Interestingly, the SLE signalling pathway which was flagged earlier also implicates autoimmune processes. SLE itself is also documented as an autoimmune disease [71] and whether increasing DM1 severity is also the result of autoimmune processes needs to be clarified.

#### 3.5.4. Additional Evidence for Immune-Related Changes in the Blood of DM1 Patients

A comprehensive analysis of blood cell composition and biochemistry has been performed in DM1 and compared with healthy control reference values [72]. In summary, an increase in white blood cell count (critical to mounting an immune response) is seen in those with DM1. Moreover, a number of biomolecules, including enzymes (such as lactate dehydrogenase, gamma-glutamyltranspeptidase, and creatine kinase), cholesterol, and hormones (luteinizing hormone), that play a role in immunity are reported to be altered. Furthermore, changes in blood enzyme activity in DM1 patients compared to healthy controls, implicate decreased superoxide dismutase and catalase activities [73]. In DM1 sera, a link between the catabolic Immunoglobin G (IgG) rate and the CTG repeat length expansion is found, whereby increased repeat length was correlated to reduced IgG levels [44]. In congenital myotonic dystrophy (CDM) muscle an upregulation of the pro-inflammatory interleukin-6 (IL-6) myokine signaling pathway is implicated but this has not been reported in adult DM1 [74]. As noted above, gene expression changes in inflammatory markers in DM1 glial cell lines [33], and genes associated with the innate immune response in DM1 [34] have previously been documented.

#### 3.5.5. Immune System Involvement in Other Muscular Dystrophies

These changes are by no means unique to DM1. Indeed, muscular health depends on the ability of the immune system to repair damage. However, in a number of muscular dystrophies, immune system changes are associated with increases in disease severity [75]. It is clear that inflammatory processes can lead to neuromuscular cell death thereby causing functional impairments [76]. Among the best studied changes examining this has been in Duchenne muscular dystrophy (DMD) which involves inflammatory processes which result in an imbalance in macrophage 1 and 2 phenotypes from the innate immune system [77]. Other muscular dystrophies such as the X-linked Becker muscular dystrophy has also been reported to involve immune system impairments [78]. Moreover, in polymyositis, an inflammatory myopathy wherein the immune system attacks the muscles and thus impairs muscle function, acts in an autoimmune manner [79]. Facioscapulohumeral muscular dystrophy (FSHD) is an autosomal dominant slowly progressive muscular dystrophy where Inflammatory changes in skeletal muscle are implicated in disease-onset [80]. The role of immune related changes in other muscular dystrophies, including limb-girdle muscular dystrophy and Emery-Dreifuss muscular dystrophy, has not yet been comprehensively studied.

### 3.6. Master Regulators

#### 3.6.1. Immune System Related Master Regulators

A number of master regulators are implicated in the immune response in the current analyses. *PRDM1* is a master upstream regulator of several processes related to immunity affecting multiple T-cell types and B-cell maturation. Moreover, there is cross-talk between the T-cell exhaustion and SLE signaling pathway as both pathways can impact autoimmunity [81,82,83]. Alterations in *PRDM1* expression may influence memory T-cell production and as a result alter the ability upon re-infection to mount an immune response. *FOXD1* is involved in autoimmunity through its regulation of *IFNγ*, *IL2*, *IL4*, and *NFAT* complexes. Of note, *FOXD1* and *FOXJ1* bind together [84]. *FOXJ1* can impair autoimmunity as it is a modulator of Th1 activation, with its deficiency resulting in autoimmunity due to a role in antagonizing NFκB activity. This regulates inflammatory responses. *CIITA* is responsible for the MHCII expression on the cell membrane playing a key role in pathogen sensing in the immune system, while another master regulator *SALL4* is an immunogenic antigen which influences HLA-DR expression. The master regulators also act to influence immunity by regulation of B lymphocytes. For example, *PAX5*, commits cells to a B-cell fate and affects secretion of IgG, a key element of the immune response. Some regulators such as *VIM* code for a receptor (vimentin) expressed on the cell surface, which is required for the attachment of pathogens. Another regulator *QKI* alters inflammation by downstream regulation of the *AHR* and NFκB signalling and as such, similar to the Fox genes can also regulate autoimmunity. *MXI1* has been implicated in *IFNα* mediated immune system activation and its dysregulation in SLE [85] while *Mxd1* regulates inflammation via Vitamin D and also alters leukocyte proliferation and therefore immunity via cross-talk with microRNA 155 [86], a microRNA which was also implicated to interact with *QXI*. While another key transcriptional regulator *BAX*, is often implicated in apoptosis, it plays an important role in innate immunity [87]. Decreased muscle mass (and diminished antigen presentation cell capacity) may be important linking elements demonstrating the interplay between muscle and immunity. 

#### 3.6.2. Muscle Related Master Regulators

Many master regulators, e.g., *FOXD1* [88], *PAX5* [89], *CIITA* [90], *QKI* [91,92,93], *VEGFA* [94], *VIM* [95], *IL4* [96], and *MLX* [97] are implicated in muscle differentiation and/or repair processes. These two processes are essential for muscle-wasting observed in DM1. Despite that expression profiles (pathway and top-levels) were measured in blood, a number of master regulators that are implicated in muscle differentiation and/or repair processes were associated with DM1 severity in this study.

Previous studies have shown that muscle degeneration observed in muscular dystrophies results from secondary pathological mechanisms that exacerbate the primary genetic defect, such as reduced blood flow, inflammation, and fibrosis [98]. This is in line with the predicted inhibition of *VEGFA* and activation of *E. coli* B4 LPS, pointing at the impairment of blood flow in muscles, leading to a lack of nutrients needed for muscle repair [94,99]. LPS is a strong inducer of many cytokines, which are important regulators of muscle protein balance [100]. Rapamycin (Sirolimus) has been previously shown to ameliorate dystrophic phenotypes in muscular dystrophy mouse models [101,102]. Rapamycin via *mTOR/mTORC1* inhibition improves immune function in the elderly, and also prevents aging of skeletal muscle [103] which is relevant in light of reports of progeria in DM1 [104]. There is a link between *BAX* as a regulator of (mitochondrial) apoptosis and muscle mass which reinforce our earlier pathway analysis and gene set / mutation analysis findings in Figure 3 and Figure 6.

### 3.7. Increased DM1 Severity in Relation to Aging as Result of Dysregulated Immunity and Muscle-Wasting

The severity of neuromuscular symptoms can be captured using the MIRS score, a five-point scale characterizing skeletal muscle function [105]. Others have demonstrated that impaired muscle function is related to the degree of muscle-wasting and failure of muscle repair processes which itself is thought to reflect accelerated cellular aging [106,107,108]. DM1 is a progeroid disease [104], characterized by age-related symptoms such as insulin resistance, impairments in leptin, and testosterone production [109]. Low levels of free testosterone and growth hormone are key features in aging DM1 male patients. This is also seen in healthy aging males which have associated reduced muscle mass [109,110,111,112]. Aging itself is associated with skeletal muscle mass loss [105,113,114] which also confers a loss of immunity as skeletal muscle acts has antigen presentation capacity. Higher muscle inflammatory vulnerability is a feature of both normal aging and DM1; however, in DM1 the gradient of muscle dystrophy is higher and muscle repair is more limited [55]; see Figure 8B. Taken together, the accelerated aging processes in DM1 may not only lead to muscle mass loss but also losses in immunity. A number of molecular mechanisms highlighted in the enriched gene set analyses here (e.g., *mTOR*, *NFAT,* and *miR-155*), may reflect muscle-wasting and increasing muscular impairment in DM1 [105]. Taken together, we speculate that muscle-wasting, accelerated cellular aging, and immunity changes are linked together resulting in increased DM1 severity.

## 4. Materials and Methods

### 4.1. RNA Isolation, Sequencing, and Differential Gene Expression (DGE) Analysis

#### 4.1.1. RNA Isolation and Sequencing

We performed RNA sequencing analysis of differential gene expression and alternative splicing in whole blood samples obtained from the preselected 30 individuals with DM1. Whole peripheral blood RNA samples were collected using Tempus Blood RNA Tubes (Life Technologies, Grand Island, NY, USA), and total RNA was extracted using MagMAX kit (Thermo Fisher Scientific, Waltham, MA, USA), according to the manufacturer’s instructions. RNA samples were further quantified using the NanoDrop 1000 Spectrophotometer and Qubit Fluorometer (Thermo Fisher Scientific, MA, USA) and RNA integrity was assessed with the 2200 TapeStation Instrument (Agilent Technologies, Santa Clara, CA, USA). Globin mRNAs and ribosomal RNAs were depleted using the Globin-Zero Gold rRNA Removal Kit (Illumina, San Diego, CA, USA). Barcoded RNA libraries were generated according to the manufacturer’s protocol using the Ion Total RNA-Seq Kit v2 and the Ion Xpress RNA-Seq barcoding kit (Thermo Fisher Scientific). The size and concentration of the libraries were assessed using the 2200 TapeStation System. Sequencing templates were prepared on the Ion Chef System using the Ion PI Hi-Q Chef Kit (Thermo Fisher Scientific). Sequencing was performed on an Ion Proton machine using an Ion PI v3 chip (Thermo Fisher Scientific) according to the manufacturer’s instructions.

#### 4.1.2. Data Analysis and Differential Expression Analysis

The FASTQC tool (http://www.bioinformatics.babraham.ac.uk/projects/fastqc/, accessed on 14 May 2021, accessed on 14 May 2021) was used to assess and summarize the quality of the raw sequence data in terms of library size, read length distribution, mean read quality distribution, mean quality for each position in the read, and base frequency for each position in the read. Reads were aligned to the reference genome (Homo sapiens, GRCh38.86, downloaded from Ensembl) (http://www.ensembl.org/, accessed on 14 May 2021) using STAR [115] and bowtie2 [116] aligners to find full and partial mappings (https://www.thermofisher.com/, accessed on 14 May 2021, the Ion Torrent RNASeqAnalysis Plugin). Alignment was analyzed by Picard tools [117] to extract read counts. Reads with at least 0.5 counts per million (cpm) across 10 samples were retained and further normalized for sample-specific effects using weighted trimmed mean of the log expression ratios (trimmed mean of M values (TMM)) method [118], accounting for sequencing depth and RNA composition bias. Principal Component Analysis (PCA) was applied on normalized gene expression values to explore the patterns present in the data. Statistical analyses (differential expression analysis) were carried out with R (www.cran.r-project.org, accessed on 14 May 2021) using R/Bioconductor [119] (www.bioconductor.org/, accessed on 14 May 2021) package “limma” with voom transformation [120], at the gene level, regressing gene expression with group status while adjusting for covariates (sex) (design model of ~0+group+sex). Only genes with *p*-value < 0.01, independent of magnitude of change, were considered as differentially expressed and used in the subsequent analyses. Heatmaps of the differentially expressed genes in both the OPTIMISTIC and DMBDI datasets were created on normalized data using the RStudio heatmap.2 function (see Supplementary Materials Script S6.1). The OPTIMISTIC histogram of the *p*-value distribution of the resulting differential gene expression (DGE) which can be found in the Supplementary Materials Script S6.2 and S6.3.

The DMBDI procedures for RNA isolation and microarray performing are described elsewhere [27]. We processed the raw CEL files using the “oligo” Bioconductor R package [121] with RMA normalization [122] and filtering out features with low median intensities (less than 4 in 6 samples). Next, we used the “limma” R package for statistical analysis of differential expression, using a linear model fit including sex for the contrast between the Group 2 and Group 1. As with OPTIMISTIC dataset, only genes with *p*-value < 0.01, independent of magnitude of change, were considered as differentially expressed and were included in further analyses. The DMBDI histogram of the *p*-value distribution of the resulting DGE was created (Supplementary Materials Script S6.4).

### 4.2. Functional Analysis

#### 4.2.1. Gene Enrichment Analysis in IPA

Using Ingenuity Pathway Analysis (IPA, version 00.06; Ingenuity Systems Inc., Redwood City, CA, USA) [123], we performed gene set enrichment analyses, in which genes and their corresponding proteins are assigned to the functional categories, ‘canonical pathways’ and ‘biofunctions’. For background expression (reference set) we used all genes detectably measured in each of the datasets.

IPA Canonical Pathway analysis identifies signaling and metabolic pathways that are most likely to be perturbed based on the differentially expressed genes in our datasets. IPA Biofunctions Analysis identifies the biological functions and diseases that are most significant to our datasets. Both methods employ a right-tailed Fisher’s Exact Test to identify statistically significant over-representation of the differentially expressed genes in a given pathway/biological function and/or disease and *p*-value of less than 0.01 was used as a cut off to indicate a non-random association.

#### 4.2.2. Causal Network Analysis in IPA (Master Regulators)

Causal Network Analysis (CNA) identifies network of upstream regulators that can work together and control the expression of dataset genes. It uses experimentally observed causal relationships between regulators and dataset genes and allows multiple interaction steps to gene expression changes. Therefore, CNA allows to detect potential novel master regulators that operate though other regulators, especially in cases where few or no relationships exist directly between it and the dataset genes. We selected the most relevant master regulators with a distance of up to two steps based on the criteria: overlap *p*-value and network bias corrected *p*-value cutoff of 0.05, and an absolute value of activation z-score above 1.5. After identification of the upstream regulators their activation or inhibition was predicted, given the observed gene expression changes in our dataset. Analysis is based on expected causal effects between upstream regulators and target genes derived from the literature compiled in the IKB. The term “upstream regulator” refers to any molecule that can affect the expression, transcription, or phosphorylation of another molecule endogenous or exogenous (drug). IPA uses a z-score algorithm to make predictions. For the detailed description of the method please refer to the following reference [123].

#### 4.2.3. Gene Enrichment Analysis in Reactome

For the gene enrichment analysis two independent gene sets were used, the OPTIMISTIC study with mRNA-sequencing data, and the DMBDI study with gene expression data. From both gene sets, genes with a *p*-value < 0.01 were selected. For the gene enrichment analysis we used the ReactomeFIPlugIn (v7.2.0) in Cytoscape (v3.7.1), which is designed to find pathways and network patterns (www.reactome.org/tools/reactome-fiviz, accessed on 14 May 2021).

Both Reactome pathway analysis and gene set/mutation analysis on a network and module level were performed. The Reactome pathway enrichment analysis is based on the hierarchical organized pathways structure similar to the Reactome web application (www.reactome.org/PathwayBrowser/, accessed on 14 May 2021). For the gene set/mutation analysis on network and module level in Reactome we used the Functional Interaction (FI) network (v2018, https://reactome.org/, accessed on 14 May 2021), a manually curated pathway-based protein functional interaction network covering human proteins, which allows the construction of a FI sub-network based on a set of genes. This FI sub-network was then used for (i) pathway enrichment for pathways originating from different databases, (Reactome, KEGG, Panther, NCI-PID, and BioCarta) and (ii) gene ontology (GO) term enrichment analysis for the three GO domains: biological process, molecular function, and cellular component. A network clustering algorithm [124] was applied on the FI sub-network, which allows pathway and gene set enrichment for both pathways and GO terms on cluster (module) level.

In the results section we show the results of the Reactome pathway analysis (Figure 5) and the GO term enrichment analysis for biological processes (Figure 6) on network level. In the Supplementary Materials S5, we also provide the results of the (i) pathway enrichment for pathways originating from the following databases: Reactome, KEGG, Panther, NCI-PID, and BioCarta, both on network and module level and (ii) GO term enrichment analysis for the three GO domains: biological processes, molecular function, and cellular component, both on network and module level.

Additionally, by analysing the gene set hierarchy, we detected the (two) top-level ancestors for the enriched Reactome and KEGG pathways, and GO terms. To find the top-level ancestors for the GO terms, only the “is a” relationship is used (http://geneontology.org/docs/ontology-relations/, accessed on 14 April 2021).

### 4.3. Splice Variant Analysis

Since the DM1 transcriptome is associated with a number of known splicing events compared to controls, splice variant analysis (described in the Supplementary Materials S7) was performed to assess if DM1 severity was also associated with changes in the same or other splice variants.

Interventionary studies involving animals or humans, and other studies that require ethical approval, must list the authority that provided approval and the corresponding ethical approval code.

### 4.4. Study Participants and Clinical Information

Study participants were recruited from the European OPTIMISTIC project, a multi-center randomized trial conducted in neuromuscular referral centers in: Nijmegen, the Netherlands; Munich, Germany; Paris, France; and Newcastle, United Kingdom; and included in the final dataset based on the flow diagram in Supplementary Materials Figure S1. Inclusion criteria for this trial were ambulatory patients with genetically confirmed DM1 who suffered from severe fatigue, as detailed elsewhere [26]. From a total of 344 patients assessed for eligibility, 89 patients were ineligible due to physical and mental disabilities and other clinical reasons (for further details [125]. Out of 255 DM1 patients recruited in the trial, we selected a sample of 30 individuals based on CTG repeat length and neuromuscular severity as ascertained by the muscular impairment rating scale (MIRS) [125]. The 30 samples were divided in three equal groups (n = 10). Group 1 consisted of participants with CTG repeat length ≤ 400 and MIRS score 1 or 2. Group 2 was defined by CTG repeat length ≤ 400 and MIRS score 3 to 5. Group 3 was defined by CTG repeat length > 400 and MIRS 3 to 5. Information on age of onset was not available for two participants, one participant in Group 1 and one participant in Group 3.

For the independent dataset we selected a subgroup using the same MIRS and CTG repeat length criteria from the *Dystrophia Myotonica* Biomarker Discovery Initiative (DMBDI) microarray dataset, as described in [27]. This resulted in data from 12 subjects selected, n = 6 in Group 1 and n = 6 in Group 2, using the same criteria as used in the OPTIMISTIC dataset. Further characterization of the individuals in the two groups are given in Table 1.

### 4.5. Ethical Approval

Prior to participation in the OPTIMISTIC study, all participants had provided written informed consent. The study was conducted in accordance with the declaration of Helsinki and approved by the medical-ethical scientific committee for human research at each of the four participating clinical centers. Ethical approval for the DMBDI dataset was already present from their participating clinical centers at LMU Munich, Germany, University of Rochester, New York, and the University of Florida Institutional Review Board, Gainesville, FL, USA.

## 5. Conclusions

Symptom severity in DM1 is associated in two independent datasets (OPTIMISTIC and DMBDI) with a transcriptional signature in blood associated with innate and adaptive immunity in gene-enrichment analyses. Whether increased DM1 severity is associated with a dysregulated immune system needs to ascertained. If true, strategies to alter immunity in DM1 may be helpful to decrease disease severity. Furthermore, it will be intriguing to see if more general regulatory inflammatory pharmacology [17] and non-pharmacological interventions improving DM1 symptoms (e.g., cognitive behavioral therapy and exercise) converge on reductions in inflammation and restoration of immunity leading to symptomatic improvement.

## Figures and Tables

**Table 1:** DM1 patient characteristics in the OPTIMISTIC and DMBDI cohorts.

	Group Size (n)	Age (Year)	Age at Disease Onset (Year)	Male	Female	MIRS (1–5)	Modal CTG Repeat Length
OPTIMISTIC gr. 1	10	48.5 (8.3)	40.2 (12.1)	4 (40%)	6 (60%)	1.9 (1–2)	179.8 (114.6)
OPTIMISTIC gr. 2	10	48.3 (2.1)	34.1 (8.1)	6 (60%)	4 (40%)	3.8 (3–5)	291.0 (71.2)
OPTIMISTIC gr. 3	10	49.0 (1.9)	27.6 (10.0) ^1^	4 (40%)	6 (60%)	3.9 (3–5)	728.5 (228.4)
DMBDI group 1	6	45.5 (14.0)	41.5 (13.2) ^2^	3 (50%)	3 (50%)	1.5 (1–2)	209.3 (252.8)
DMBDI group 2	6	46.3 (9.5)	22.7 (13.6)	2 (33.3)	4 (66.7%)	3.2 (3–5)	252.8 (77.1)

Dara are mean (SD), n (%), ^1^ One missing value for age at disease onset, ^2^ Two missing values for age at disease onset, MIRS (range).

## Data Availability

Researchers wishing to access the transcriptomic data performed in the context of the OPTIMISTIC study are requested to contact JCG (Jeffrey.Glennon@ucd.ie) and sign a data access agreement. Access to the dataset pertaining to the MariGold DMDBI study are requested to contact the MariGold Foundation, Canada.

## Supplementary Materials

The following are available online at https://www.mdpi.com/article/10.3390/ijms23063081/s1.
