# Supplementary material for: Blood Transcriptome Profiling Links Immunity to Disease Severity in Myotonic Dystrophy Type 1 (DM1)
Source: Int J Mol Sci. 2022 Mar 12;23(6):3081. doi: 10.3390/ijms23063081 (PMC8954763; doi:10.3390/ijms23063081)
Supplement: Supplementary file 1 [file ijms-23-03081-s001.zip › ijms-1556026 supplementary.pdf]

International Journal of *Molecular Sciences*

MDPI

Supplementary Materials

S1. Patient selection criteria analysis

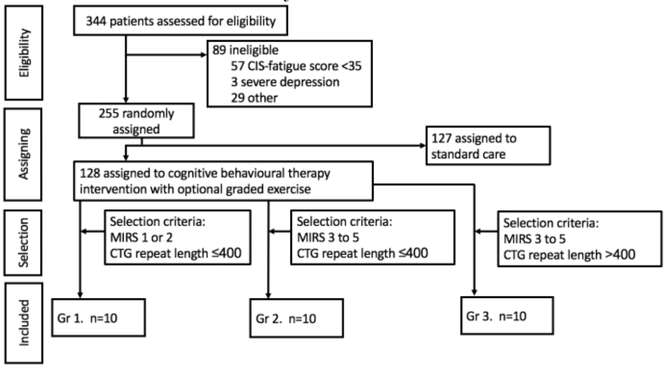

Figure S1. DM1 patient flow diagram showing inclusion / exclusion criteria to reach the final sample number of 10 DM1 patient participants for the groups 1, 2 and 3 [1].

S2. R-studio analysis

S2.1. Exploratory analysis principal component analysis (PCA)

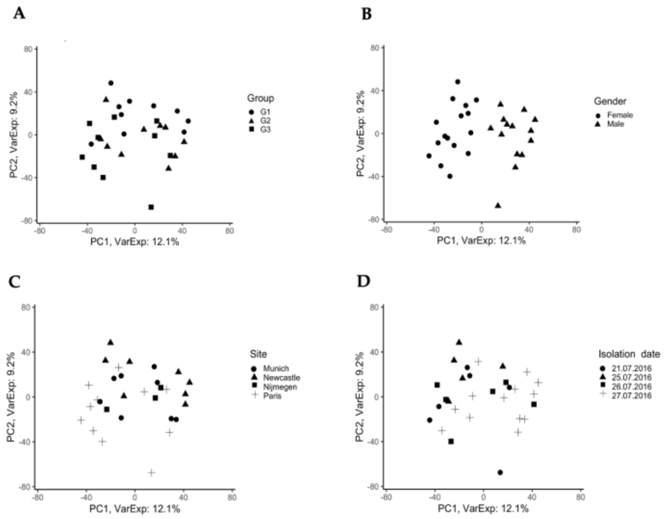

Figure S2.1. Principal Component Analysis (PCA). **A**, No effect was found across groups on PC1 and PC2; **B**, gender was associated with a distinct distribution in two dimensional space between PC1 and PC2; **C**, there was no impact of the clinical EU site (Newcastle, Nijmegen, Paris, and Munich) on PC1 and PC2; **D**, there was no effect of the time of isolation on PC1 and PC2.

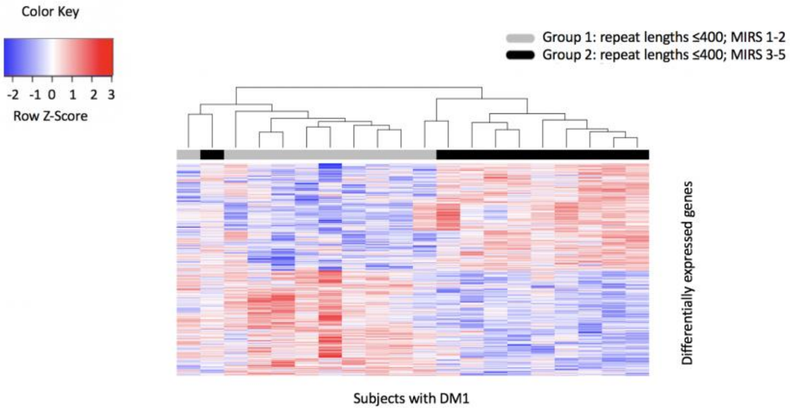

Figure S2.2. Hierarchical Clustering Heatmap of all 683 differentially expressed genes in OPTIMISTIC DM1 group 1 and group 2 patients. The color indicates the differentially expressed genes, red, higher expressed genes, blue, lower expressed genes. The heatmap presents significant differentially expressed genes (P-value ≤ 0.01) in

subjects with DM1 from group 1 (CTG repeat length ≤400, MIRS score 1 - 2) compared to group 2 (CTG repeat length ≤400, MIRS score 3 - 5).

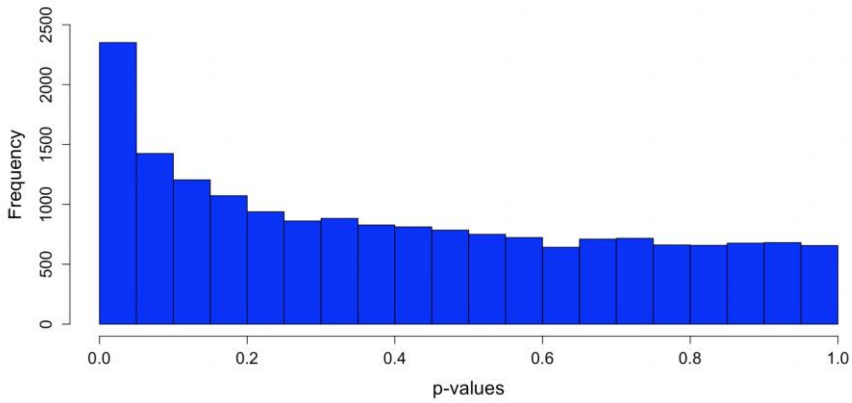

Figure S2.3. P-value distribution of differential expression analysis of OPTIMISTIC Group 2 (CTG repeat length ≤400, MIRS score 3 - 5) versus Group 1 (CTG repeat length ≤400, MIRS score 1 - 2) RNAseq data (Voom followed by Limma) per comparison.

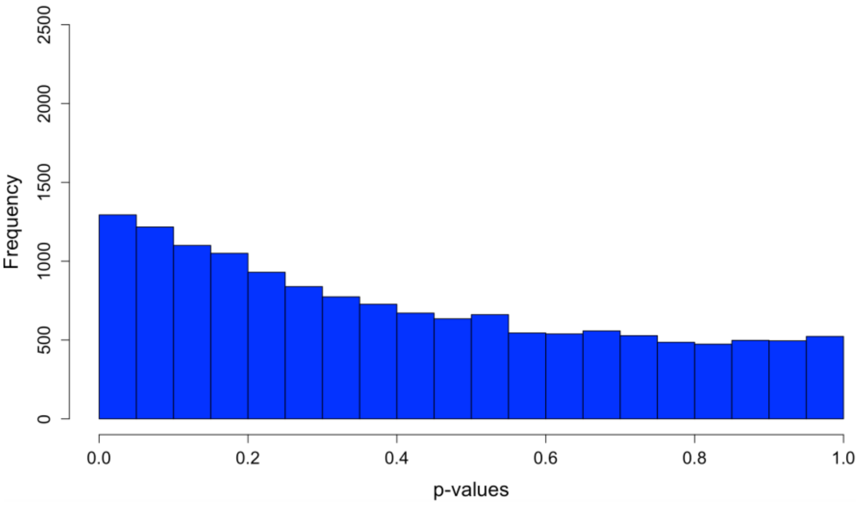

Figure S2.4. P-value distribution of differential expression analysis of DMBDI microarray data (Limma) per comparison G2 (CTG repeat length ≤400, MIRS score 3 - 5) versus G1 (CTG repeat length ≤400, MIRS score 1 - 2) RNAseq data (Voom followed by Limma) per comparison.

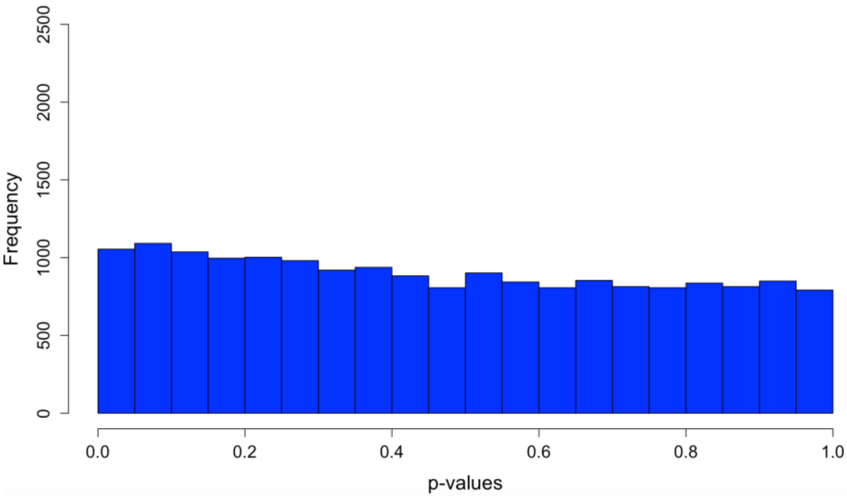

Figure S2.5. P-value distribution of differential expression analysis of OPTIMISTIC Group 3 (CTG repeat length >400, MIRS score 3 - 5) compared to Group 2 (CTG repeat length ≤400, MIRS score 3 - 5) RNAseq data (Voom followed by Limma) per comparison.

Splice variant analysis

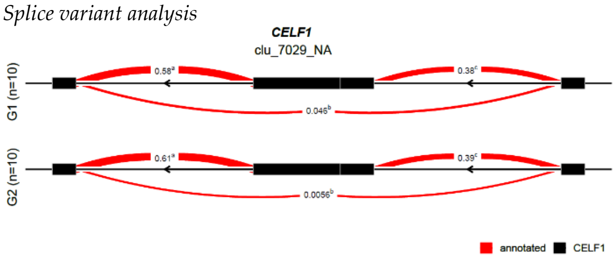

	chr	start	end	verdict	dPSI
a	chr11	47482856	47483453	annotated	0.024
b	chr11	47482856	47484389	annotated	−0.023
c	chr11	47483532	47484389	annotated	0.000

Figure S2.6. Sashimi plot showing the percentage of exon:exon junctions spanning reads supporting the inclusion and exclusion of exon 5 in the *CELF1* gene (CCDS7938).

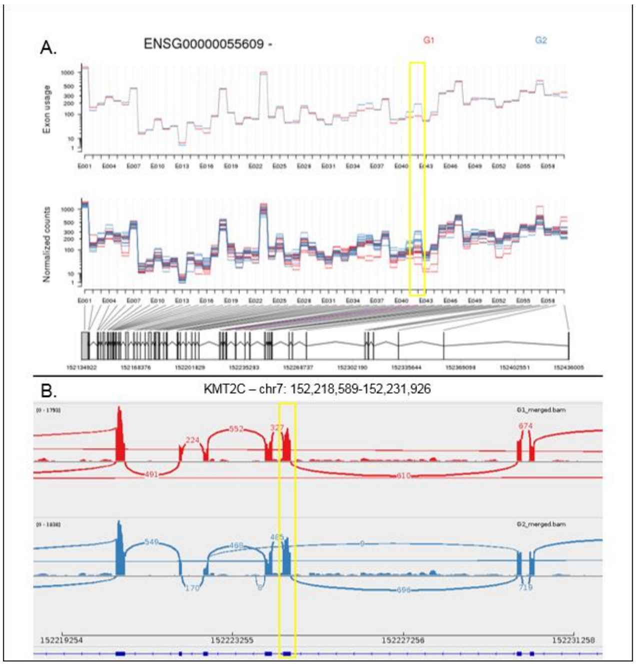

Figure S2.7. Visualization of the *KMT2C* gene using DEXSeq and Sashimi, showing differences in exon usage between Group 1 and Group 2. **A.** Visualization of the differential exon usage for the KMT2C gene using DEXSeq. Exon bin E042 (GChR38 coordinates: chr7: 152224435-152224616), highlighted by the yellow rectangle, shows a significant difference in exon usage, with an adjusted P-value of 3.59e-06 and a log2 fold change of 1.08. Exon usage panel: expression estimates fitted by the generalized linear model, based on the number of reads per exon bin, after subtraction of overall changes in gene expression; Normalized counts panel: count values for each sample, based on the number of reads per exon bin, normalized by dividing it by the size factors, making the counts comparable between samples; Bottom: flattened gene model. Significant exon bins are shown in purple. **B.** Sashimi plot of a part of the *KMT2C* gene, highlighting exon bin E042 (see A.) with the yellow rectangle (chr7: 152,224,435-152,224,616). (Red) Data for Group 1; (blue) Group 2.

S3. Methods Ingenuity pathway analysis (IPA)

S3.1. Canonical Pathway Analysis

Canonical pathways analysis was used to identify signaling and metabolic pathways that were most significant to the set of differentially expressed genes between Group 2 and Group 1. The strength of the association is measured by a ratio, calculated by dividing the number of genes from the dataset that map to the pathway by the total number of molecules that make up that pathway and that are in the reference set. The confidence of the association between our dataset and the canonical pathway is measured with a P-value of overlap, determining the probability that the association between the genes in the dataset and the canonical pathway is explained by chance alone. Moreover, the overall activation/inhibition states of canonical pathways are predicted based on a z-score algorithm 107], which is used to compare our dataset with the canonical pathway patterns. The canonical pattern is calculated taking into account the activation state of one of more key molecules when the pathway is activated and also the molecules' causal relationships with each other (*i.e.*, activation edge and the inhibition

edge between the molecules based on literature findings) to generate an activity pattern for the molecules and also the end-point functions in the pathway.

S3.2. Diseases and biological functions

The Diseases and Functions Analysis aims to identify the biological functions and/or diseases that are most relevant to our dataset. We explored which diseases and cellular processes are predicted to be increasing or decreasing based on the pattern of differentially expressed genes in our dataset and what genes are driving these directional changes.

S3.3. Upstream regulators and causal networks

We used Upstream Regulator Analysis to identify the upstream regulators and predict whether they are activated or inhibited, given the observed gene expression changes in our dataset. Analysis is based on expected causal effects between upstream regulators and target genes derived from the literature compiled in the IKB. The term "upstream regulator" refers to any molecule that can affect the expression, transcription, or phosphorylation of another molecule. IPA uses a z-score algorithm to make predictions [2].

S4. Gene set clustering and filtering approaches

Clustering of gene sets and genes

To facilitate the interpretation of the four graphs we have clustered both gene sets and genes, using the following technique.

- Calculate pairwise distance between observations by means of Euclidean metric.
- Estimation of hierarchical clustering information by means of furthest distance.

The dendrograms on the bottom and right side of the genes heatmap show the clustering hierarchy for both genes and gene sets respectively. For clustering the genes, we have used the genes heatmap. The gene sets have been clustered with the top-level ancestor information (and NOT the genes heatmap).

Filtering of gene sets and genes

For both GO Biological processes (BP) and IPA diseases and functions (DF), the number of genes sets and genes is large. To decrease the size of these graphs we have used filters for the gene sets and genes. The filter parameters and their values for both BP and DF are found here: https://rdrr.io/github/jmw86069/jamenrich/man/mem_gene_path_heatmap.html

Table S4.1.

Filter parameter	Description	BP	DF
Gene set filtering			
min_set_ct	minimum number of genes required for each set, all other sets are excluded	1	2
min_set_ct_each	minimum number of genes required for each set, required for at least one enrichment test	2	4
Gene filtering			
min_gene_ct	minimum number of occurrences of each gene across the gene sets, all other genes are excluded.	2	4

The order of operations for filtering is as follows:

- The gene set filters are applied before filtering genes, in order to ensure all genes are present from the start.
- The genes are filtered after gene set filtering, in order to remove gene sets which were not deemed "significant" based upon the required number of genes.

S5. Methods Reactome

Overview of DM1 analysis method

The DM1 analysis method is shown in Figure S5.1. The DM1 analysis is based on two independent gene sets, being the OPTIMISTIC study with mRNA-sequencing data, and the DMBDI study with gene expression data. From these two gene sets the following genes are selected: OPTIMISTIC genes with P-value<0.01, and DMBDI genes with P-value<1. The selected genes are used as input for a gene enrichment analysis, using the Reactome pathway analysis and the Gene set/mutation analysis, in the Reactome FI app in CytoScape. During the Gene set/mutation analysis, a network diagram can be made. The results of the gene enrichment analysis are transferred to an excel-file "DM1 enrichment results" for further analysis.

Figure S5.1. shows an overview of the DM1 analysis method. An explanation of the different blocks can be found below:

- Gray blocks represent manual/programmed actions by the analyst.
- Green block represents an excel-sheet in an excel-file named "DM1 Gene Analysis"
- White blocks represent groups of cells in the different excel sheets. The text in the white block is also used to annotate the groups of cells.
- Purple blocks represent additional excel-files which are used and/or created during the analysis.
- Yellow blocks represent manual actions within CytoScape with the ReactomeFIV-app installed.
- Blue blocks represent databases and resources used during the DM1 analysis.

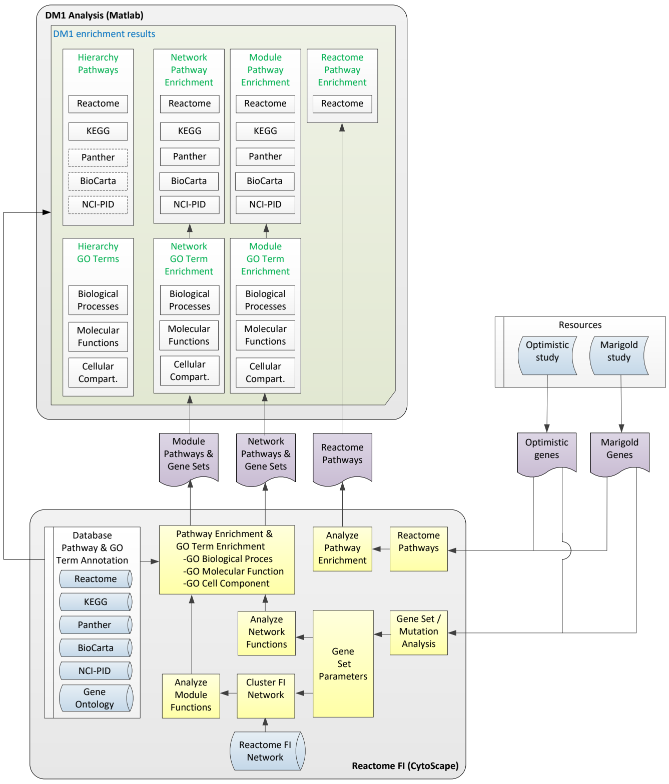

Figure S5.1. Overview of the DM1 analysis method.

Preparation of OPTIMISTIC and DMBDI data

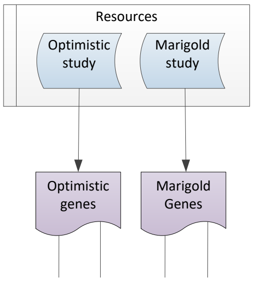

Figure S5.2. Preparation of OPTIMISTIC and DMBDI data.

The method to prepare the OPTIMISTIC and DMBDI data is described elsewhere. See main paper, section "Data analysis and differential expression analysis".

The results are exported to the following files:

- OPTIMISTIC genes (P<0.01):
  - OPTIMISTIC.G2vsG1.xlsx
- DMBDI genes (P<1):
  - DMBDI.G2vsG1.xlsx

Gene enrichment analysis

The gene enrichment analysis is done using the Reactome FI app within CytoScape.

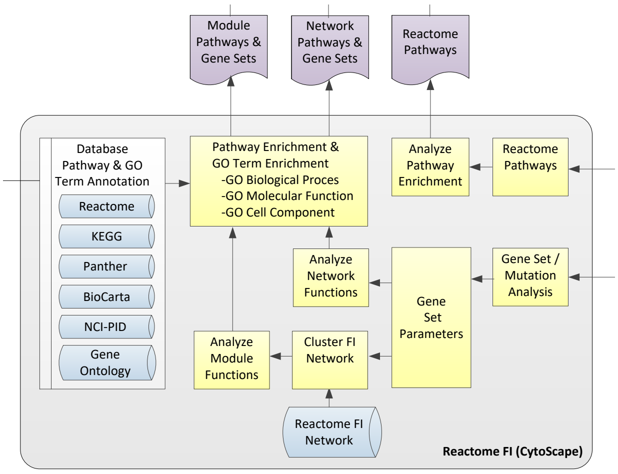

Figure S5.3. Gene enrichment analysis.

The yellow blocks in Figure S5.3. describe the use of this app.

The gene enrichment analysis results in a number of tables, which can be exported as tab-delimited files via a right-mouse-click in the tables. The exported files are described below.

Reactome Pathways > Analyse Pathway Enrichment
Exported text file:
- Tree PW.txt

The text file above contains a list with Reactome pathways. For each of the Reactome pathways the following parameters are shown:
- Ratio of protein in pathway: for ratios of numbers of genes contained in pathways to total genes in the Reactome FI network
- Number of proteins in pathway: for numbers of genes in pathways
- Protein from gene set: for numbers of hit genes from the query gene list
- P-value: calculated based on binomial test
- Failure discovery rate (FDR): calculated based on p-values using Benjamini-Hocherberg method
- Hit genes: in the pathway

Gene Set/Mutation Analysis > Gene Set Parameters > Analyze Network Functions
Exported text files:
- Pathway Enrichment: Network PW.txt
- GO (Term Enrichment) Biological Processes: Network BP.txt
- GO (Term Enrichment) Molecular Functions: Network MF.txt
- GO (Term Enrichment) Cell Component: Network CC.txt

The text files above contain a list with gene sets (or pathways). For each of the gene sets the following parameters are shown:
- Ratio of protein in gene set (pathway): for ratios of numbers of genes contained in pathways to total genes in the Reactome FI network
- Number of proteins in gene set: for numbers of genes in pathways
- Protein from network, for numbers of hit genes from the query gene list
- P-value, calculated based on binomial test
- Failure discovery rate (FDR): calculated based on p-values using Benjamini-Hocherberg method
- Nodes: hit genes in the gene set (pathway)

Gene Set/Mutation Analysis > Gene Set Parameters > Analyze Module Functions
Exported text files:
- Pathway Enrichment: Modules PW.txt
- GO (Term Enrichment) Biological Processes: Modules BP.txt
- GO (Term Enrichment) Molecular Functions: Modules MF.txt
- GO (Term Enrichment) Cell Component: Modules CC.txt

The text files above contain a list with gene sets (pathways). For each of the gene sets the following parameters are shown:

- Module: the module number
- Ratio of protein in gene set (pathway): for ratios of numbers of genes contained in pathways to total genes in the Reactome FI network
- Number of proteins in gene set: for numbers of genes in pathways
- Protein from module, for numbers of hit genes from the query gene list
- P-value, calculated based on binomial test
- Failure discovery rate (FDR): calculated based on p-values using Benjamini-Hocherberg method
- Nodes: hit genes in the gene set (pathway)

The Node Table created during the Gene Set/Mutation Analysis can be exported via the CytoScape menu items: File > Export > Table to File …
Exported text file:

- ipa_p.lt.0.01.txt default node.csv

Presentation of DM1 enrichment results

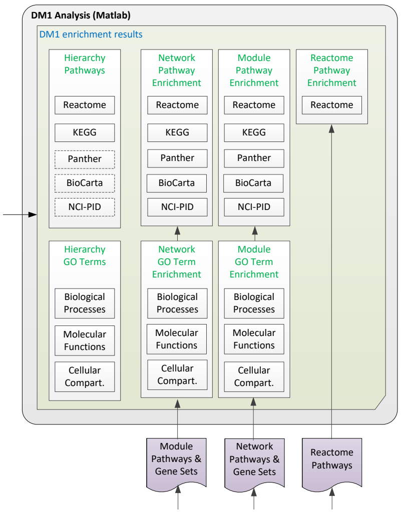

Figure S5.4. Presentation of DM1 enrichment results

All DM1 enrichment results described in the previous section are stored in an excel-file "DM1 enrichment results" with the help of a Matlab-script "DM1 Analysis". Figure S4.4., shows the structure of the excel-file. The excel-file has two main sections with pathways enrichment results and GO term enrichment results.

The text below describes the:

- Structure and data sources of the excel file.
- Detailed description of the enrichment data.
- Filtering of the enrichment data.

Structure and data sources of the excel file

The pathway enrichment section of the excel file has five sub-sections for the Reactome, KEGG, Panther, BioCarta and NCI-PID data.

For the Reactome data we use the same hierarchical pathway structure as used by the Reactome pathway analysis of the ReactomeFIViz-app. An example is shown in the graph below.

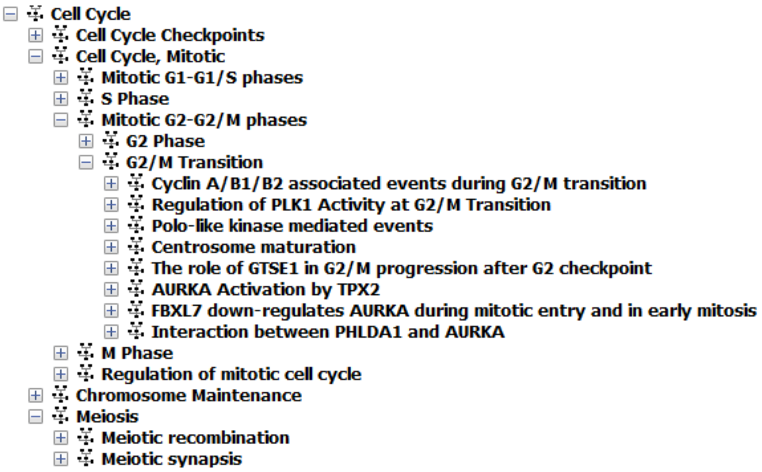

Figure S5.5. Part of the Reactome pathway hierarchy.

The Reactome pathway hierarchy (tree) is reconstructed (excluding the reactions) in the excel-file as shown in Table S5.6. (for the original excel-files see Reference [6]), which is similar to the section of the Reactome pathway hierarchy shown in Figure S5.5.

Table S5.6. Reconstruction of the Reactome pathway hierarchy

	A	B	C	D	E–M	N
1	Hierarchy Pathways and GO Terms					
2	Level	Pathway / GO Term (Database)	Level 1 Pathway / GO Term	Level 2 Pathway / GO Term	Level >=3 Pathway / GO Term	Enriched (highest level) Pathway / GO Term
3	0	PW(Reactome)				
4	1	PW(Reactome)	Cell Cycle			Cell Cycle
5	2	PW(Reactome)	Cell Cycle	Meiosis		Meiosis
6	3	PW(Reactome)	Cell Cycle	Meiosis	Meiotic synapsis	Meiotic synapsis
7	3	PW(Reactome)	Cell Cycle	Meiosis	Meiotic recombinatio	Meiotic recombination
8	2	PW(Reactome)	Cell Cycle	Cell Cycle, Mitotic		Cell Cycle, Mitotic
9	3	PW(Reactome)	Cell Cycle	Cell Cycle, Mitotic	Mitotic G2-G2/M phas	Mitotic G2-G2/M phases
10	4	PW(Reactome)	Cell Cycle	Cell Cycle, Mitotic	G2 Phase	G2 Phase
11	4	PW(Reactome)	Cell Cycle	Cell Cycle, Mitotic	G2/M Transition	G2/M Transition
12	5	PW(Reactome)	Cell Cycle	Cell Cycle, Mitotic	Polo-like kinase	Polo-like kinase mediated events
13	5	PW(Reactome)	Cell Cycle	Cell Cycle, Mitotic	Regulation of PL	Regulation of PLK1 Activity at G2/M Transition
14	5	PW(Reactome)	Cell Cycle	Cell Cycle, Mitotic	Centrosome ma	Centrosome maturation
15	6	PW(Reactome)	Cell Cycle	Cell Cycle, Mitotic	Recruitment	Recruitment of mitotic centrosome proteins and complexes
16	6	PW(Reactome)	Cell Cycle	Cell Cycle, Mitotic	Loss of protei	Loss of proteins required for interphase microtubule organization from the centrosome
17	7	PW(Reactome)	Cell Cycle	Cell Cycle, Mitotic	Loss of Nlp	Loss of Nlp from mitotic centrosomes
18	5	PW(Reactome)	Cell Cycle	Cell Cycle, Mitotic	Cyclin A/B1/B2 a	Cyclin A/B1/B2 associated events during G2/M transition
19	6	PW(Reactome)	Cell Cycle	Cell Cycle, Mitotic	Phosphorylat	Phosphorylation of proteins involved in the G2/M transition by Cyclin A:Cdc2 complexes
20	5	PW(Reactome)	Cell Cycle	Cell Cycle, Mitotic	The role of GTSE	The role of GTSE1 in G2/M progression after G2 checkpoint
21	5	PW(Reactome)	Cell Cycle	Cell Cycle, Mitotic	FBXL7 down-reg	FBXL7 down-regulates AURKA during mitotic entry and in early mitosis
22	5	PW(Reactome)	Cell Cycle	Cell Cycle, Mitotic	AURKA Activatic	AURKA Activation by TPX2
23	5	PW(Reactome)	Cell Cycle	Cell Cycle, Mitotic	Interaction betw	Interaction between PHLDA1 and AURKA

For each of the pathways the following items are shown:

- Level
  - The level of the pathway
  - Level 0 is the database source of the pathways, in this case the Reactome database
- Pathway / GO Term (Database)
  - Database source of the pathways, in this case the Reactome database
- Level 1 Pathway / GO Term
  - Highest level pathway
- Level 2 Pathway / GO Term
  - Next highest level pathway
- Level >=3 Pathway / GO Term
  - Higher level pathways
- Enriched (highest level) Pathway / GO Term
  - Enriched (highest level) pathway found by the enrichment analysis of the ReactomeFIviz app.

The Reactome pathway hierarchy (tree) is reconstructed with the help of the files shown below.

- Reactome top level pathways
  - Manually created input file, contains top level pathways, which form start of Reactome tree search
- Reactome pathways
  - Data file: https://reactome.org/download/current/ReactomePathways.txt
  - Origin: https://reactome.org/download-data > Pathways > Complete List of Pathways
- Reactome pathway genes
  - Data file: https://reactome.org/download/current/ReactomePathways.gmt.zip
  - Origin: https://reactome.org/download-data > Specialized data formats > Reactome Pathways Gene Set
- Reactome pathway hierarchy
  - Data file: https://reactome.org/download/current/Complex_2_Pathway_human.txt
  - Origin: https://reactome.org/download-data > Pathways > Pathways hierarchy relationship

The top level pathways are used a starting point for a forward tree search. The result is stored in the Hierarchy Pathways columns of the excel file (see Figure S4.4.)

The KEGG pathway hierarchy (tree) can also be reconstructed as shown below. The KEGG pathway hierarchy has only three levels

- KEGG pathway hierarchy
  - Load https://www.genome.jp/kegg/pathway.html#organismal
    Select KEGG pathway structure
    Copy/paste text into sheet 'KEGG' of excel-file 'TreeKEGG.xlsx'
    Save excel-file 'TreeKEGG.xlsx' for use in gene enrichment analysis
    Run matlab-script 'readkegg'

The Panther, BioCarta and NCI-PID pathways have no hierarchical structure, but form a simple list of pathways: that is why they are drawn as a dotted line in Figure S4.4. These pathways are extracted from the databases shown below.

- Panther pathways
  - Data file: https://amp.pharm.mssm.edu/static/hdfs/harmonizome/data/panther/attribute_list_entries.txt.gz
  - Origin: https://amp.pharm.mssm.edu/Harmonizome/about > PANTHER > Pathways > Attribute List
- Panther pathway genes
  - Data file: https://amp.pharm.mssm.edu/static/hdfs/harmonizome/data/panther/gene_attribute_edges.txt.gz
  - Origin: https://amp.pharm.mssm.edu/Harmonizome/about > PANTHER > Pathways > Gene-Attribute Edge List
- NCI-PID pathways
  - Data file: https://amp.pharm.mssm.edu/static/hdfs/harmonizome/data/pid/attribute_list_entries.txt.gz
  - Origin: https://amp.pharm.mssm.edu/Harmonizome/about > Pathway Interaction Database > Pathways > Attribute List
- NCI-PID pathway genes
  - Data file: https://amp.pharm.mssm.edu/static/hdfs/harmonizome/data/pid/gene_attribute_edges.txt.gz"
  - Origin: https://amp.pharm.mssm.edu/Harmonizome/about > Pathway Interaction Database > Pathways > Gene-Attribute Edge List
- BioCarta pathways
  - Data file: https://amp.pharm.mssm.edu/static/hdfs/harmonizome/data/biocarta/attribute_list_entries.txt.gz
  - Origin: https://amp.pharm.mssm.edu/Harmonizome/about > Biocarta > Pathways > Attribute List
- BioCarta pathway genes
  - Data file: https://amp.pharm.mssm.edu/static/hdfs/harmonizome/data/biocarta/gene_attribute_edges.txt.gz
  - Origin: https://amp.pharm.mssm.edu/Harmonizome/about > Biocarta > Pathways > Gene-Attribute Edge List

The pathway enrichment results are then added to the excel-file as described below.

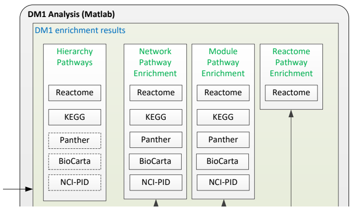

Figure S5.7. Section with pathways enrichment results in excel-file

The Reactome pathway enrichment results of the Gene Set/Mutation Analysis are stored in the following columns of the excel file:

- Pathways network level (Network PW.txt): Network Pathway Enrichment
- Pathways module level (Modules PW.txt): Module Pathway Enrichment

The Reactome pathway enrichment results of the Reactome Pathway analysis, are stored in the following columns of the excel file:

- Pathway enrichment (Tree PW.txt): Reactome Pathway Enrichment

The enriched pathways of the different databases are projected on the hierarchical structure of Reactome and KEGG, and the list structure of Panther, BioCarta and NCI-PID databases.

Using the hierarchy of the pathways as a basis for the presentation, enables projection of pathway enrichment results of multiple gene sets (such OPTIMISTIC and DMBDI) to be projected on one structure. Grouping and/or clustering of pathway enrichment results from the one or more gene sets becomes clearly visible.

The GO term enrichment section of the excel file has three sub-sections for the GO domains Biological Processes, Molecular Functions and Cell Components.

Also the GO terms are hierarchically organized. A section of the reconstructed Gene Ontology domain "Biological Processes" is shown in Table S5.8. (for the original excel-file see Reference [6]). For each of the biological processes a series of items is shown. A description of these items can be found below Table S5.6.

Table S5.8. A section of the reconstruction of the Gene Ontology domain "Biological Processes"

	A	B	C	D	E–M	N
1	Hierarchy Pathways and GO Terms					
2	Level	Pathway / GO Term (Database)	Level 1 Pathway / GO Term	Level 2 Pathway / GO Term	Level >=3 Pathway / GO Term	Enriched (highest level) Pathway / GO Term
3243	1	BP(GO)	biological adhesion			biological adhesion
3244	2	BP(GO)	biological adhesion	adhesion of symbiont to host		adhesion of symbiont to host
3245	2	BP(GO)	biological adhesion	cell adhesion		cell adhesion
3246	2	BP(GO)	biological adhesion	intermicrovillar adhesion		intermicrovillar adhesion
3247	2	BP(GO)	biological adhesion	multicellular organism adhesion		multicellular organism adhesion
3248	1	BP(GO)	biological phase			biological phase
3249	2	BP(GO)	biological phase	cell cycle phase		cell cycle phase
3250	2	BP(GO)	biological phase	estrous cycle phase		estrous cycle phase
3251	2	BP(GO)	biological phase	hair cycle phase		hair cycle phase
3252	2	BP(GO)	biological phase	menstrual cycle phase		menstrual cycle phase
3253	2	BP(GO)	biological phase	single-celled organism vegetative grow		single-celled organism vegetative growth phase
3254	1	BP(GO)	biological regulation			biological regulation
3255	2	BP(GO)	biological regulation	negative regulation of phosphatidylcho		negative regulation of phosphatidylcholine metabolic process
3256	2	BP(GO)	biological regulation	regulation of biological process		regulation of biological process
3257	2	BP(GO)	biological regulation	regulation of negative regulation of neg		negative regulation of guanyl-nucleotide exchange factor activity
3258	5	BP(GO)	biological regulation	regulation of negative regulation of neg	negative regulation o	negative regulation of cell-cell adhesion mediated by integrin
3259	6	BP(GO)	biological regulation	regulation of negative regulation of neg	positive regulation of	positive regulation of leukocyte adhesion to vascular endothelial cell
3260	5	BP(GO)	biological regulation	regulation of negative regulation of neg	negative regulation o	negative regulation of stress fiber assembly
3261	6	BP(GO)	biological regulation	regulation of negative regulation of neg	regulation of mitotic	regulation of G2/M transition of mitotic cell cycle

The GO term hierarchy is reconstructed with the help of the file shown below.

- Gene Ontology hierarchy
  - Data file: http://purl.obolibrary.org/obo/go/go-basic.obo
  - Origin: http://geneontology.org/docs/download-ontology/

The GO term hierarchy for the three GO domains is stored in the Hierarchy GO Terms columns of the excel file (see Figure S5.9.).

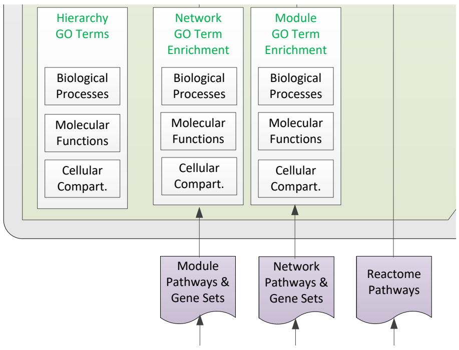

Figure S5.9. Section with GO term enrichment results in excel-file

The GO term enrichment results of the Gene Set/Mutation Analysis are stored in the following columns of the excel file (see Figure S5.9.).

- Biological process network level (Network BP.txt): Network GO Term Enrichment
- Molecular function network level (Network MF.txt): Network GO Term Enrichment
- Cell component network level (Network CC.txt): Network GO Term Enrichment
- Biological process module level (Module BP.txt): Module GO Term Enrichment
- Molecular function module level (Module MF.txt): Module GO Term Enrichment
- Cell component module level (Module CC.txt): Module GO Term Enrichment

The enriched GO terms found by the Gene Set/Mutation Analysis shown above, are projected on the hierarchical GO term structure.

Also, here the GO term hierarchy enables projection of GO term enrichment results of multiple gene sets (such OPTIMISTIC and DMBDI) to be projected on one structure.

It is important to note that the Gene Ontology hierarchy is very large. It is therefore chosen to only show the first tree levels of the complete GO hierarchy, and show the complete branch (of the tree) for the gene sets, found by the enrichment algorithm of Reactome. Of these gene sets only the 50 most dominant gene sets for every GO domain (biological process, molecular function and cell component), are projected on the GO domain hierarchy, based on their P-value.

Detailed description of the enrichment data

Network pathway and GO term enrichment
For each of the gene sets (pathways) the following parameters are shown:

- Gene set repeats in tree
  - For the Reactome and KEGG pathways, the pathways found by the enrichment analysis can be located in multiple branches (gene set repeats in tree) of the Reactome and KEGG hierarchy.
  - Also, for the GO domains, the gene sets found by the enrichment analysis can be located in multiple branches (gene set repeats in tree) of the GO domain hierarchy.
  - In the column you can find the following values: a(b).
    - a: is the number of gene set repeats in the tree.
    - b: is the number of unique ancestors in the tree, where unique means that the (two) top level ancestors of the repeating gene set are different.
- Unique ancestors in tree
  - This column has a value 1 when the gene set has two unique top level ancestors (also see: gene set repeats in tree).
  - This column can be used to filter out all gene sets with unique ancestors.
- Ratio of protein in gene set
  - Ratio of numbers of genes contained in pathways to total genes in the Reactome FI network
- Number of proteins in gene set
  - Numbers of genes in pathways
- Protein from network
  - Numbers of hit genes from the query gene list
- P-value from gene set
  - Calculated based on binomial test
  - Can be used to filter gene sets (pathways) based on P-value
- Failure discovery rate (FDR) from gene set
  - Calculated based on p-values using Benjamini-Hocherberg method
  - Can be used to filter gene sets (pathways) based on FDR
- Maximum abs(logFC) nodes
  - Max(abs(logFC)) of all hit genes in the gene set (pathway)
  - Also see: Nodes: a±b
  - Can be used to filter gene sets (pathways) based on fold change
- Maximum –log10(P) nodes
  - Max(-log10(P)) of all hit genes in the gene set (pathway)
  - Also see: Nodes: a±b
  - Can be used to filter gene sets (pathways) based on P-value
- Nodes:a±b
  - Nodes are hits genes in the gene set (pathway)
  - a: is equal to round(-log10(P)), where P is the P-value of the node (gene)
  - ±b: is equal to round(abs(logFC)), where logFC is the log(FoldChange) of the node (gene)

Module pathway and GO term enrichment

The text files above contain a list with gene sets (pathways). For each of the gene sets the following parameters are shown:

- Modules: the module number
  - A gene set (pathway) can be present in multiple modules, which are shown in this column.
- Main module: the module number
  - The module number for which the P-value of the gene set (pathway) has the smallest value.
- Number of repeats in tree
  - Described earlier
- Unique ancestors in tree
  - Described earlier
- Ratio of protein in gene set
  - Described earlier
- Number of proteins in gene set
  - Described earlier
- Protein from module
  - Numbers of hit genes from the query gene list
- P-value from gene set
  - Described earlier
- Failure discovery rate (FDR) from gene set
  - Described earlier
- Maximum abs(logFC) ModNodes
  - Described earlier
- Maximum –log10(P) ModNodes
  - Described earlier
- Module(P-value gene set) Nodes :a±b
  - The genes of all modules are shown here, organized per module. For each module the P-value of the gene set is shown). For each node (gene) the P-value and fold change is shown as described earlier.

Reactome pathway enrichment

The text file above contains a list with Reactome pathways. For each of the Reactome pathways the following parameters are shown:

- Number of repeats in tree
  - Described earlier
- Unique ancestors in tree
  - Described earlier
- Ratio of protein in pathway
  - Ratio of numbers of genes contained in pathways to total genes in the Reactome FI network
- Number of proteins in pathway
  - Number of genes in pathway
- Protein from gene set
  - Number of hit genes from the query gene list
- P-value from pathway
  - Calculated based on binomial test
  - Can be used to filter gene pathways based on P-value
- Failure discovery rate (FDR) from pathway
  - Described earlier
- Maximum abs(logFC) nodes
  - Described earlier
- Maximum –log10(P) nodes
  - Described earlier
- Nodes:a±b
  - Described earlier

Filtering of the enrichment data

To filter the enrichment data:

- Set filters on row 2 using: Data > Filter
- Before setting a new filter, clear all old filters: Home > Sort & Filter > Clear
- If unexpected results are seen reload excel-file, sometimes it is difficult to return to the initial unsorted state of the excel file.
- Use of the sorting option causes mixing of the Pathway / Go Term hierarchy.
- Pushing the Group/Ungroup buttons 1,2,3,4 in the top-left corner of the excel sheet, will hide/unhide part of the columns, which facilitates ease copying of sections of the excel-file. It is advised to always push button 4 before pushing buttons 1,2,3, to prevent unexpected results.

A number of filtering examples and its results are shown below, for the original excel-files see Reference [6].

Network Pathway / Go Term enrichment for filter settings:

- Pathway / Go Term (Database) [column B]: PW (KEGG)
- P-value From Gene Set [column Y]: <0.05

Table S5.10.

Hierarchy Pathways and GO Terms					Network Pathway / GO Term Enrichment									
Level	Pathway / GO Term (Database)	Level 1 Pathway / GO Term	Level 2 Pathway / GO Term	Enriched (highest level) Pathway / GO Term	NumberOf Repeats InTree	Unique Ancestors InTree	RatioOf Proteins InGeneSe	NumberOf Proteins InGeneSet	Proteins From Networ	P-value From GeneS	FDR From GeneS	Maximum abs(logFC) Nodes	Maximum -10log(P) Nodes	Nodes:-10log(P)±log(FC)
3	PW(KEGG)	Metabolism	Global and overview maps	Biosynthesis of amino acids	1 (1)	1	0,0066	75	5	3,67E-02	3,98E-01	1,0	3,7	PFKL:4-0, SHMT2:3-0, GLUL:2+0, ALDOA:2-0, ARG2:2+1
3	PW(KEGG)	Metabolism	Glycan biosynthesis and metabolism	N-Glycan biosynthesis	1 (1)	1	0,0043	49	4	3,20E-02	3,98E-01	0,6	3,0	MOGS:3-0, DDOST:2-0, MAN2A2:2+0, DPM2:2+1
3	PW(KEGG)	Genetic Information Prc	Replication and repair	Base excision repair	1 (1)	1	0,0029	33	3	4,67E-02	3,98E-01	0,5	3,1	POLD1:3-0, MPG:3-0, NTHL1:2-1
3	PW(KEGG)	Environmental Informat	Signal transduction	Notch signaling pathway	1 (1)	1	0,0042	48	5	6,59E-03	3,66E-01	0,6	3,9	APH1A:4-0, LFNG:3-0, DTX4:3-1, MFNG:3-0, DVL1:2-0
3	PW(KEGG)	Cellular Processes	Transport and catabolism	Phagosome	1 (1)	1	0,0134	152	10	4,30E-03	3,09E-01	1,2	3,3	HLA-DOA:3-1, FCGR2C:3+1, FCGR1A:3+1, HLA-DPA1:3-1, TUBB6:3-1, TUBB4A:2-
3	PW(KEGG)	Cellular Processes	Transport and catabolism	Lysosome	1 (1)	1	0,0109	123	7	3,12E-02	3,98E-01	1,0	3,4	GNPTG:3-0, NEU1:3-0, CLTA:3-0, LAMP1:2-0, MAN2B1:2-0, AP1M2:2+1, NAGPA
3	PW(KEGG)	Cellular Processes	Cell growth and death	Apoptosis	1 (1)	1	0,012	136	7	4,89E-02	3,98E-01	0,9	2,9	JUN:3-1, LMNB2:2-0, HRK:2-1, DIABLO:2+0, BAD:2-0, MAP2K1:2-0, FADD:2-0
3	PW(KEGG)	Cellular Processes	Cell growth and death	Ferroptosis	1 (1)	1	0,0035	40	4	1,68E-02	3,98E-01	0,6	3,3	PCBP2:3+0, FTH1:2+1, SLC3A2:2-0, MAP1LC3B:2+0
3	PW(KEGG)	Organismal Systems	Immune system	Hematopoietic cell lineage	1 (1)	1	0,0086	97	6	3,17E-02	3,98E-01	1,0	3,3	HLA-DOA:3-1, FCGR1A:3+1, HLA-DPA1:3-1, HLA-DMB:2-0, HLA-DRA:2-0, CD1A:2
3	PW(KEGG)	Organismal Systems	Immune system	Th17 cell differentiation	1 (1)	1	0,0095	107	6	4,70E-02	3,98E-01	0,6	3,3	HLA-DOA:3-1, JUN:3-1, AHR:3-1, HLA-DPA1:3-1, HLA-DMB:2-0, HLA-DRA:2-0
3	PW(KEGG)	Organismal Systems	Immune system	Intestinal immune network for IgA production	1 (1)	1	0,0043	49	5	7,17E-03	3,66E-01	1,1	3,3	HLA-DOA:3-1, HLA-DPA1:3-1, CCR10:2-1, HLA-DMB:2-0, HLA-DRA:2-0
3	PW(KEGG)	Human Diseases	Immune disease	Asthma	1 (1)	1	0,0027	31	4	7,15E-03	3,66E-01	0,6	3,3	HLA-DOA:3-1, HLA-DPA1:3-1, HLA-DMB:2-0, HLA-DRA:2-0
3	PW(KEGG)	Human Diseases	Immune disease	Autoimmune thyroid disease	1 (1)	1	0,0047	53	4	4,07E-02	3,98E-01	0,6	3,3	HLA-DOA:3-1, HLA-DPA1:3-1, HLA-DMB:2-0, HLA-DRA:2-0
3	PW(KEGG)	Human Diseases	Immune disease	Inflammatory bowel disease (IBD)	1 (1)	1	0,0058	65	5	2,17E-02	3,98E-01	0,6	3,3	HLA-DOA:3-1, JUN:3-1, HLA-DPA1:3-1, HLA-DMB:2-0, HLA-DRA:2-0
3	PW(KEGG)	Human Diseases	Immune disease	Allograft rejection	1 (1)	1	0,0034	38	4	1,42E-02	3,98E-01	0,6	3,3	HLA-DOA:3-1, HLA-DPA1:3-1, HLA-DMB:2-0, HLA-DRA:2-0
3	PW(KEGG)	Human Diseases	Immune disease	Graft-versus-host disease	1 (1)	1	0,0036	41	4	1,82E-02	3,98E-01	0,6	3,3	HLA-DOA:3-1, HLA-DPA1:3-1, HLA-DMB:2-0, HLA-DRA:2-0
3	PW(KEGG)	Human Diseases	Neurodegenerative disease	Alzheimer disease	1 (1)	1	0,0151	171	10	9,43E-03	3,98E-01	0,8	3,9	APH1A:4-0, NDUFAB1:3-0, NDUFS7:2-0, NDUFV1:2-0, NDUFC1:2-0, NDUFB5:2-(
3	PW(KEGG)	Human Diseases	Neurodegenerative disease	Parkinson disease	1 (1)	1	0,0126	142	8	2,33E-02	3,98E-01	0,9	3,0	NDUFAB1:3-0, SLC25A6:3-0, NDUFS7:2-0, NDUFV1:2-0, UBB:2+1, NDUFC1:2-0,
3	PW(KEGG)	Human Diseases	Neurodegenerative disease	Huntington disease	1 (1)	1	0,0171	193	10	2,01E-02	3,98E-01	0,5	3,0	NDUFAB1:3-0, CLTA:3-0, SLC25A6:3-0, NDUFS7:2-0, AP2B1:2+0, POLR2E:2-0, NI
3	PW(KEGG)	Human Diseases	Endocrine and metabolic disease	Type I diabetes mellitus	1 (1)	1	0,0038	43	4	2,12E-02	3,98E-01	0,6	3,3	HLA-DOA:3-1, HLA-DPA1:3-1, HLA-DMB:2-0, HLA-DRA:2-0
3	PW(KEGG)	Human Diseases	Infectious disease: bacterial	Staphylococcus aureus infection	1 (1)	1	0,005	56	7	4,84E-04	9,84E-02	1,4	3,9	C1QB:4+1, HLA-DOA:3-1, FCGR2C:3+1, FCGR1A:3+1, HLA-DPA1:3-1, HLA-DMB:2
3	PW(KEGG)	Human Diseases	Infectious disease: bacterial	Tuberculosis	1 (1)	1	0,0158	179	12	1,57E-03	1,90E-01	1,0	3,3	HLA-DOA:3-1, FCGR2C:3+1, FCGR1A:3+1, HLA-DPA1:3-1, RAB5B:2+0, LAMP1:2-(
3	PW(KEGG)	Human Diseases	Infectious disease: viral	Human T-cell leukemia virus 1 infection	1 (1)	1	0,0194	219	10	4,19E-02	3,98E-01	0,6	3,3	RANBP1:3-0, HLA-DOA:3-1, JUN:3-1, SLC25A6:3-0, HLA-DPA1:3-1, HLA-DMB:2-(
3	PW(KEGG)	Human Diseases	Infectious disease: viral	Influenza A	1 (1)	1	0,0151	171	10	9,43E-03	3,98E-01	0,7	3,3	HLA-DOA:3-1, JUN:3-1, SLC25A6:3-0, IRF9:3+1, HLA-DPA1:3-1, MAP2K3:3+1, HL
3	PW(KEGG)	Human Diseases	Infectious disease: viral	Epstein-Barr virus infection	1 (1)	1	0,0178	201	11	1,05E-02	3,98E-01	0,6	3,3	HLA-DOA:3-1, JUN:3-1, IRF9:3+1, HLA-DPA1:3-1, MAP2K3:3+1, VIM:2-0, PSMD3
3	PW(KEGG)	Human Diseases	Infectious disease: parasitic	Toxoplasmosis	1 (1)	1	0,01	113	7	2,10E-02	3,98E-01	0,6	3,3	HLA-DOA:3-1, HLA-DPA1:3-1, MAP2K3:3+1, IRAK1:2-0, HLA-DMB:2-0, HLA-DRA:
3	PW(KEGG)	Human Diseases	Infectious disease: parasitic	Leishmaniasis	1 (1)	1	0,0065	74	8	5,04E-04	9,84E-02	1,0	3,3	HLA-DOA:3-1, FCGR2C:3+1, JUN:3-1, FCGR1A:3+1, HLA-DPA1:3-1, IRAK1:2-0, HL

Network Pathway / Go Term enrichment for filter settings:

- Pathway / Go Term (Database) [column B]: PW(Panther), PW(BioCarta), PW(NCI-PID)
- P-value From Gene Set [column Y]: <0.05

Table S5.11.

Hierarchy Pathways and GO Terms					Network Pathway / GO Term Enrichment									
Level	Pathway / GO Term (Database)	Level 1 Pathway / GO Term	Level 2 Pathway / GO Term	Enriched (highest level) Pathway / GO Term	NumberOf Repeats InTree	Unique Ancestors InTree	RatioOf Proteins InGeneSe	NumberOf Proteins InGeneSet	Proteins From Networ	P-value From GeneS	FDR From GeneS	Maximum abs(logFC) Nodes	Maximum -10log(P) Nodes	Nodes:-10log(P)±log(FC)
4	PW(Panther)			T cell activation	1 (1)	1	0,0072	81	7	3,87E-03	3,09E-01	0,6	4,2	LCP2:4+0, HLA-DOA:3-1, JUN:3-1, HLA-DPA1:3-1, HL/
4	PW(Panther)			FAS signaling pathway	1 (1)	1	0,0029	33	3	4,67E-02	3,98E-01	0,6	2,9	JUN:3-1, LMNB2:2-0, FADD:2-0
4	PW(BioCarta)			er associated degradation (erad) pathway	1 (1)	1	0,001	11	3	2,54E-03	2,47E-01	0,4	3,0	MOGS:3-0, MAN2B1:2-0, MAN2A2:2+0
4	PW(BioCarta)			oxidative stress induced gene expression via nrf2	1 (1)	1	0,0012	13	2	4,00E-02	3,98E-01	0,6	2,9	JUN:3-1, AKR7A2:3-0
4	PW(BioCarta)			cadmium induces dna synthesis and proliferation in macrophages	1 (1)	1	0,0012	13	2	4,00E-02	3,98E-01	0,6	2,9	JUN:3-1, MAP2K1:2-0
4	PW(BioCarta)			angiotensin ii mediated activation of jnk pathway via pyk2 dependent signaling	1 (1)	1	0,0029	33	3	4,67E-02	3,98E-01	0,6	3,1	MEF2C:3-0, JUN:3-1, MAP2K1:2-0
4	PW(NCI-PID)			Trk receptor signaling mediated by the MAPK pathway	1 (1)	1	0,0029	33	3	4,67E-02	3,98E-01	0,6	3,1	MEF2C:3-0, MAP2K3:3+1, MAP2K1:2-0
4	PW(NCI-PID)			RAC1 signaling pathway	1 (1)	1	0,0048	54	4	4,31E-02	3,98E-01	0,6	2,9	JUN:3-1, PIP5K1B:3+1, MAP2K3:3+1, NCKAP1:3+1
4	PW(NCI-PID)			JNK signaling in the CD4+ TCR pathway	1 (1)	1	0,0012	14	2	4,57E-02	3,98E-01	0,6	4,2	LCP2:4+0, JUN:3-1
4	PW(NCI-PID)			Validated nuclear estrogen receptor alpha network	1 (1)	1	0,0055	62	5	1,81E-02	3,98E-01	0,6	2,9	JUN:3-1, MPG:3-0, CD82:2-0, NCOA1:2+0, DDX54:2-(
4	PW(NCI-PID)			HIF-1-alpha transcription factor network	1 (1)	1	0,0058	65	5	2,17E-02	3,98E-01	0,6	3,7	PFKL:4-0, CITED2:3-0, JUN:3-1, ALDOA:2-0, NCOA1:2
4	PW(NCI-PID)			Signaling mediated by p38-gamma and p38-delta	1 (1)	1	0,001	11	2	2,95E-02	3,98E-01	0,6	2,6	MAP2K3:3+1, STMN1:2-0

Network Pathway / Go Term enrichment for filter settings:

- Pathway / Go Term (Database) [column B]: BP(GO)
- P-value From Gene Set [column Y]: < 0.007

Table S5.12.

Hierarchy Pathways and GO Terms					Network Pathway / GO Term Enrichment									
Level	Pathway / GO Term (Database)	Level 1 Pathway / GO Term	Level 2 Pathway / GO Term	Enriched (highest level) Pathway / GO Term	NumberOf Repeats InTree	Unique Ancestors InTree	RatioOf Proteins InGeneSe	NumberOf Proteins InGeneSet	Proteins From Networ	P-value From GeneS	FDR From GeneS	Maximum abs(logFC) Nodes	Maximum -10log(P) Nodes	Nodes:-10log(P)±log(FC)
2	BP(GO)	biological regulation	regulation of negative	negative regulation of guanyl-nucleotide exchange factor activity	9 (2)	1	0,0003	4	2	5,34E-03	2,56E-01	0,6	2,2	GPSM1:2-1, STMN1:2-0
5	BP(GO)	biological regulation	regulation of negative	negative regulation of cell-cell adhesion mediated by integrin	10 (1)	1	0,0002	2	2	1,38E-03	2,56E-01	0,7	2,8	SWAP70:3-0, WNK1:2+1
5	BP(GO)	biological regulation	regulation of negative	negative regulation of stress fiber assembly	19 (1)	1	0,0013	17	4	1,24E-03	2,56E-01	0,6	3,7	CLASP1:4+0, PPP1R9A:2+1, STMN1:2-0, INPP5K:2+0
6	BP(GO)	biological regulation	regulation of negative	interferon-gamma-mediated signaling pathway	2 (2)	1	0,0053	70	7	3,12E-03	2,56E-01	2,5	3,5	FCGR1B:4+1, IRF8:3-0, IRF9:3+1, FCGR1A:3+1, HLA-D
5	BP(GO)	biological regulation	regulation of negative	positive regulation of skeletal muscle cell differentiation	26 (1)	1	0,0002	3	2	3,06E-03	2,56E-01	0,4	3,5	ARNTL:4+0, MEF2C:3-0
10	BP(GO)	biological regulation	regulation of negative	activation of cysteine-type endopeptidase activity	46 (5)	1	0,0011	14	3	6,63E-03	2,56E-01	0,3	2,2	IFI16:2+0, BAD:2-0, FADD:2-0
7	BP(GO)	biological regulation	regulation of biologic	acetylcholine catabolic process in synaptic cleft	25 (7)	1	0,0002	2	2	1,38E-03	2,56E-01	1,7	3,2	COLQ:3+1, ACHE:2+2
9	BP(GO)	biological regulation	regulation of molecul	activation of cysteine-type endopeptidase activity	46 (5)	1	0,0011	14	3	6,63E-03	2,56E-01	0,3	2,2	IFI16:2+0, BAD:2-0, FADD:2-0
6	BP(GO)	biological regulation	regulation of molecul	negative regulation of guanyl-nucleotide exchange factor activity	9 (2)	1	0,0003	4	2	5,34E-03	2,56E-01	0,6	2,2	GPSM1:2-1, STMN1:2-0
7	BP(GO)	cellular component orga	cellular component o	mitochondrial respiratory chain complex I assembly	8 (2)	1	0,0046	61	7	1,46E-03	2,56E-01	0,5	3,0	NDUFAB1:3-0, NDUFS7:2-0, NDUFV1:2-0, NDUFC1:2
7	BP(GO)	cellular component orga	cellular component o	peptide antigen assembly with MHC class II protein complex	4 (2)	1	0,0002	3	2	3,06E-03	2,56E-01	0,5	2,4	HLA-DMB:2-0, HLA-DRA:2-0
5	BP(GO)	cellular component orga	cellular component o	chromatin assembly	4 (2)	1	0,0006	8	3	1,39E-03	2,56E-01	0,4	2,4	CHAF1B:2+0, GATAD1:2-0, NOC2L:2-0
7	BP(GO)	cellular process	cellular component o	mitochondrial respiratory chain complex I assembly	8 (2)	1	0,0046	61	7	1,46E-03	2,56E-01	0,5	3,0	NDUFAB1:3-0, NDUFS7:2-0, NDUFV1:2-0, NDUFC1:2
7	BP(GO)	cellular process	cellular component o	peptide antigen assembly with MHC class II protein complex	4 (2)	1	0,0002	3	2	3,06E-03	2,56E-01	0,5	2,4	HLA-DMB:2-0, HLA-DRA:2-0
5	BP(GO)	cellular process	cellular component o	chromatin assembly	4 (2)	1	0,0006	8	3	1,39E-03	2,56E-01	0,4	2,4	CHAF1B:2+0, GATAD1:2-0, NOC2L:2-0
6	BP(GO)	cellular process	cellular developmenta	monocyte differentiation	6 (2)	1	0,0013	17	4	1,24E-03	2,56E-01	0,6	3,1	MEF2C:3-0, JUN:3-1, PDE1B:2+1, IFI16:2+0
6	BP(GO)	cellular process	cellular metabolic pro	RNA metabolic process	10 (5)	1	0,0033	43	5	6,48E-03	2,56E-01	0,4	3,3	PCBP2:3+0, GTF2F1:3-0, POLR2E:2-0, HNRNPA0:2-0,
6	BP(GO)	cellular process	cellular metabolic pro	acetylcholine catabolic process in synaptic cleft	25 (7)	1	0,0002	2	2	1,38E-03	2,56E-01	1,7	3,2	COLQ:3+1, ACHE:2+2
5	BP(GO)	cellular process	signal transduction	interferon-gamma-mediated signaling pathway	2 (2)	1	0,0053	70	7	3,12E-03	2,56E-01	2,5	3,5	FCGR1B:4+1, IRF8:3-0, IRF9:3+1, FCGR1A:3+1, HLA-D
6	BP(GO)	developmental process	cellular developmenta	monocyte differentiation	6 (2)	1	0,0013	17	4	1,24E-03	2,56E-01	0,6	3,1	MEF2C:3-0, JUN:3-1, PDE1B:2+1, IFI16:2+0
5	BP(GO)	immune system process	antigen processing an	antigen processing and presentation of exogenous peptide antigen via MHC class II	4 (1)	1	0,0073	97	9	1,41E-03	2,56E-01	2,5	3,3	ACTR1B:3-0, HLA-DOA:3-1, CLTA:3-0, HLA-DPA1:3-1,
2	BP(GO)	immune system process	immune response	immune response	1 (2)	1	0,0285	376	22	6,33E-04	2,56E-01	2,5	4,2	LCP2:4+0, FCGR1B:4+1, VPREB3:3-1, HLA-DOA:3-1, F
6	BP(GO)	localization	cellular localization	protein targeting to mitochondrion	15 (3)	1	0,002	27	4	6,39E-03	2,56E-01	0,4	2,8	SLC25A6:3-0, TIMM13:3-0, TIMM44:3-0, TOMM40:2
10	BP(GO)	localization	establishment of loca	protein targeting to mitochondrion	15 (3)	1	0,002	27	4	6,39E-03	2,56E-01	0,4	2,8	SLC25A6:3-0, TIMM13:3-0, TIMM44:3-0, TOMM40:2
8	BP(GO)	localization	macromolecule locali	protein targeting to mitochondrion	15 (3)	1	0,002	27	4	6,39E-03	2,56E-01	0,4	2,8	SLC25A6:3-0, TIMM13:3-0, TIMM44:3-0, TOMM40:2
6	BP(GO)	metabolic process	catabolic process	acetylcholine catabolic process in synaptic cleft	25 (7)	1	0,0002	2	2	1,38E-03	2,56E-01	1,7	3,2	COLQ:3+1, ACHE:2+2
6	BP(GO)	metabolic process	cellular metabolic pro	RNA metabolic process	10 (5)	1	0,0033	43	5	6,48E-03	2,56E-01	0,4	3,3	PCBP2:3+0, GTF2F1:3-0, POLR2E:2-0, HNRNPA0:2-0,
6	BP(GO)	metabolic process	cellular metabolic pro	acetylcholine catabolic process in synaptic cleft	25 (7)	1	0,0002	2	2	1,38E-03	2,56E-01	1,7	3,2	COLQ:3+1, ACHE:2+2
5	BP(GO)	metabolic process	hormone metabolic p	acetylcholine catabolic process in synaptic cleft	25 (7)	1	0,0002	2	2	1,38E-03	2,56E-01	1,7	3,2	COLQ:3+1, ACHE:2+2
5	BP(GO)	metabolic process	nitrogen compound m	acetylcholine catabolic process in synaptic cleft	25 (7)	1	0,0002	2	2	1,38E-03	2,56E-01	1,7	3,2	COLQ:3+1, ACHE:2+2
6	BP(GO)	metabolic process	nitrogen compound m	RNA metabolic process	10 (5)	1	0,0033	43	5	6,48E-03	2,56E-01	0,4	3,3	PCBP2:3+0, GTF2F1:3-0, POLR2E:2-0, HNRNPA0:2-0,
8	BP(GO)	metabolic process	nitrogen compound m	activation of cysteine-type endopeptidase activity	46 (5)	1	0,0011	14	3	6,63E-03	2,56E-01	0,3	2,2	IFI16:2+0, BAD:2-0, FADD:2-0
7	BP(GO)	metabolic process	nitrogen compound m	protein quality control for misfolded or incompletely synthesized proteins	4 (3)	1	0,0008	11	3	3,41E-03	2,56E-01	0,3	2,7	LONP1:3-0, CLPP:2-0, YME1L1:2-0
6	BP(GO)	metabolic process	organic substance me	acetylcholine catabolic process in synaptic cleft	25 (7)	1	0,0002	2	2	1,38E-03	2,56E-01	1,7	3,2	COLQ:3+1, ACHE:2+2
8	BP(GO)	metabolic process	organic substance me	activation of cysteine-type endopeptidase activity	46 (5)	1	0,0011	14	3	6,63E-03	2,56E-01	0,3	2,2	IFI16:2+0, BAD:2-0, FADD:2-0
7	BP(GO)	metabolic process	organic substance me	protein quality control for misfolded or incompletely synthesized proteins	4 (3)	1	0,0008	11	3	3,41E-03	2,56E-01	0,3	2,7	LONP1:3-0, CLPP:2-0, YME1L1:2-0
6	BP(GO)	metabolic process	organic substance me	RNA metabolic process	10 (5)	1	0,0033	43	5	6,48E-03	2,56E-01	0,4	3,3	PCBP2:3+0, GTF2F1:3-0, POLR2E:2-0, HNRNPA0:2-0,
5	BP(GO)	metabolic process	primary metabolic prc	RNA metabolic process	10 (5)	1	0,0033	43	5	6,48E-03	2,56E-01	0,4	3,3	PCBP2:3+0, GTF2F1:3-0, POLR2E:2-0, HNRNPA0:2-0,
7	BP(GO)	metabolic process	primary metabolic prc	activation of cysteine-type endopeptidase activity	46 (5)	1	0,0011	14	3	6,63E-03	2,56E-01	0,3	2,2	IFI16:2+0, BAD:2-0, FADD:2-0
6	BP(GO)	metabolic process	primary metabolic prc	protein quality control for misfolded or incompletely synthesized proteins	4 (3)	1	0,0008	11	3	3,41E-03	2,56E-01	0,3	2,7	LONP1:3-0, CLPP:2-0, YME1L1:2-0
2	BP(GO)	response to stimulus	immune response	immune response	1 (2)	1	0,0285	376	22	6,33E-04	2,56E-01	2,5	4,2	LCP2:4+0, FCGR1B:4+1, VPREB3:3-1, HLA-DOA:3-1, F

Reactome Pathway Enrichment for filter settings:

- Pathway / Go Term (Database) [column B] = PW(Reactome)
- P-value From Pathway [column BM] < 0.05
- Maximum abs(logFC) Nodes [column BO] > 1
- Maximum –log10(P) Hitgenes [column BP] > 2.5

Table S5.13.

Hierarchy Pathways and GO Terms					Reactome Pathway Enrichment									
Level	Pathway / GO Term (Database)	Level 1 Pathway / GO Term	Level 2 Pathway / GO Term	Enriched (highest level) Pathway / GO Term	NumberOf Repeats InTree	Unique Ancestors InTree	RatioOf Protein InPathwa	NumberOf Protein InPathwa	Protein From GeneSe	P-value From Pathwa	FDR From Pathwa	Maximum abs(logFC) Hitgenes	Maximum -10log(P) Hitgenes	Hitgenes:-10log(P)±log(FC)
3	PW(Reactome)	Cell Cycle	Cell Cycle, Mitotic	Mitotic G2-G2/M phases	1 (1)	1	0,0205	177	10	2,30E-02	3,45E-01	1,2	3,7	CLASP1:4+0, TUBB4A:2+1, PSMD3:2-0, CEP131:2-0, UBB:2+1, T
4	PW(Reactome)	Cell Cycle	Cell Cycle, Mitotic	G2/M Transition	1 (1)	1	0,0202	175	10	2,15E-02	3,45E-01	1,2	3,7	CLASP1:4+0, TUBB4A:2+1, PSMD3:2-0, CEP131:2-0, UBB:2+1, T
5	PW(Reactome)	Cell Cycle	Cell Cycle, Mitotic	Regulation of PLK1 Activity at G2/M Transition	1 (1)	1	0,0101	87	7	9,92E-03	2,94E-01	1,2	3,7	CLASP1:4+0, TUBB4A:2+1, CEP131:2-0, UBB:2+1, OFD1:2+0, SS
5	PW(Reactome)	Cell Cycle	Cell Cycle, Mitotic	Centrosome maturation	1 (1)	1	0,0091	79	8	1,52E-03	2,18E-01	1,2	3,7	CLASP1:4+0, TUBB4A:2+1, CEP131:2-0, TUBGCP2:2-0, MZT2A:
6	PW(Reactome)	Cell Cycle	Cell Cycle, Mitotic	Recruitment of mitotic centrosome proteins and complexes	1 (1)	1	0,0091	79	8	1,52E-03	2,18E-01	1,2	3,7	CLASP1:4+0, TUBB4A:2+1, CEP131:2-0, TUBGCP2:2-0, MZT2A:
6	PW(Reactome)	Cell Cycle	Cell Cycle, Mitotic	Loss of proteins required for interphase microtubule organization from the centrosome	1 (1)	1	0,008	69	6	1,17E-02	2,94E-01	1,2	3,7	CLASP1:4+0, TUBB4A:2+1, CEP131:2-0, OFD1:2+0, SSNA1:2-0,
7	PW(Reactome)	Cell Cycle	Cell Cycle, Mitotic	Loss of Nlp from mitotic centrosomes	1 (1)	1	0,008	69	6	1,17E-02	2,94E-01	1,2	3,7	CLASP1:4+0, TUBB4A:2+1, CEP131:2-0, OFD1:2+0, SSNA1:2-0,
5	PW(Reactome)	Cell Cycle	Cell Cycle, Mitotic	AURKA Activation by TPX2	1 (1)	1	0,0083	72	6	1,41E-02	3,27E-01	1,2	3,7	CLASP1:4+0, TUBB4A:2+1, CEP131:2-0, OFD1:2+0, SSNA1:2-0,
4	PW(Reactome)	Cell Cycle	Cell Cycle, Mitotic	Mitotic Prometaphase	1 (1)	1	0,0202	175	10	2,15E-02	3,45E-01	1,2	3,7	CLASP1:4+0, RANGAP1:3-0, TUBB4A:2+1, CEP131:2-0, TUBGCF
5	PW(Reactome)	Cell Cycle	Cell Cycle, Mitotic	Recruitment of NuMA to mitotic centrosomes	1 (1)	1	0,0093	80	8	1,64E-03	2,18E-01	1,2	3,7	CLASP1:4+0, TUBB4A:2+1, CEP131:2-0, TUBGCP2:2-0, MZT2A:
4	PW(Reactome)	Immune System	Cytokine Signaling in I	Interferon gamma signaling	1 (1)	1	0,0088	76	7	4,92E-03	2,80E-01	2,5	3,5	FCGR1B:4+1, IRF8:3-0, IRF9:3+1, FCGR1A:3+1, HLA-DPA1:3-1, I
2	PW(Reactome)	Immune System	Adaptive Immune Syst	Adaptive Immune System	1 (1)	1	0,0694	600	25	2,07E-02	3,45E-01	2,5	4,2	LCP2:4+0, FCGR1B:4+1, ACTR1B:3-0, HLA-DOA:3-1, CLTA:3-0, I
4	PW(Reactome)	Immune System	Adaptive Immune Syst	Phosphorylation of CD3 and TCR zeta chains	1 (1)	1	0,0034	29	3	4,45E-02	3,51E-01	2,5	2,6	HLA-DPA1:3-1, HLA-DQB2:3-3, HLA-DRA:2-0
4	PW(Reactome)	Immune System	Adaptive Immune Syst	Translocation of ZAP-70 to Immunological synapse	1 (1)	1	0,003	26	3	3,40E-02	3,51E-01	2,5	2,6	HLA-DPA1:3-1, HLA-DQB2:3-3, HLA-DRA:2-0
4	PW(Reactome)	Immune System	Adaptive Immune Syst	Generation of second messenger molecules	1 (1)	1	0,0045	39	4	2,20E-02	3,45E-01	2,5	4,2	LCP2:4+0, HLA-DPA1:3-1, HLA-DQB2:3-3, HLA-DRA:2-0
3	PW(Reactome)	Immune System	Adaptive Immune Syst	MHC class II antigen presentation	1 (1)	1	0,0109	94	9	1,15E-03	2,18E-01	2,5	3,3	ACTR1B:3-0, HLA-DOA:3-1, CLTA:3-0, HLA-DPA1:3-1, HLA-DQB
4	PW(Reactome)	Immune System	Adaptive Immune Syst	PD-1 signaling	1 (1)	1	0,0035	30	4	9,27E-03	2,94E-01	2,5	2,6	HLA-DPA1:3-1, CD274:3+1, HLA-DQB2:3-3, HLA-DRA:2-0
2	PW(Reactome)	Immune System	Innate Immune Syster	Innate Immune System	1 (1)	1	0,1155	998	38	1,83E-02	3,45E-01	1,4	4,2	LCP2:4+0, C1QB:4+1, PFKL:4-0, PCBP2:3+0, ACTR1B:3-0, NEU1
5	PW(Reactome)	Metabolism	Metabolism of lipids	Synthesis of PC	1 (1)	1	0,0024	21	3	1,97E-02	3,45E-01	1,7	2,5	STARD7:3-0, ACHE:2+2, LPIN2:2+0
3	PW(Reactome)	Organelle biogenesis an	Cilium Assembly	Anchoring of the basal body to the plasma membrane	1 (1)	1	0,0112	97	7	1,70E-02	3,45E-01	1,2	3,7	CLASP1:4+0, TUBB4A:2+1, CEP131:2-0, C2CD3:2+0, OFD1:2+0,

Reactome Pathway Enrichment for filter settings:

- Pathway / Go Term (Database) [column B]: PW(Reactome)
- Unique Ancestors In Tree [column BI]: 1
- P-value From Pathway [column BM]: < 0.05
- Maximum –log10(P) Hitgenes [column BO]: > 3

Table S5.14.

Hierarchy Pathways and GO Terms					Reactome Pathway Enrichment									
Level	Pathway / GO Term (Database)	Level 1 Pathway / GO Term	Level 2 Pathway / GO Term	Enriched (highest level) Pathway / GO Term	NumberOf Repeats InTree	Unique Ancestors InTree	RatioOf Protein InPathwa	NumberOf Protein InPathwa	Protein From GeneSe	P-value From Pathwa	FDR From Pathwa	Maximum abs(logFC) Hitgenes	Maximum -10log(P) Hitgenes	Hitgenes:-10log(P)±log(FC)
3	PW(Reactome)	Cell Cycle	Cell Cycle, Mitotic	Mitotic G2-G2/M phases	1 (1)	1	0,0205	177	10	2,30E-02	3,45E-01	1,2	3,7	CLASP1:4+0, TUBB4A:2+1, PSMD3:2-0, CEP131:2-0, UBB:2
4	PW(Reactome)	Cell Cycle	Cell Cycle, Mitotic	G2/M Transition	1 (1)	1	0,0202	175	10	2,15E-02	3,45E-01	1,2	3,7	CLASP1:4+0, TUBB4A:2+1, PSMD3:2-0, CEP131:2-0, UBB:2
5	PW(Reactome)	Cell Cycle	Cell Cycle, Mitotic	Regulation of PLK1 Activity at G2/M Transition	1 (1)	1	0,0101	87	7	9,92E-03	2,94E-01	1,2	3,7	CLASP1:4+0, TUBB4A:2+1, CEP131:2-0, UBB:2+1, OFD1:2+
5	PW(Reactome)	Cell Cycle	Cell Cycle, Mitotic	Centrosome maturation	1 (1)	1	0,0091	79	8	1,52E-03	2,18E-01	1,2	3,7	CLASP1:4+0, TUBB4A:2+1, CEP131:2-0, TUBGCP2:2-0, MZ
6	PW(Reactome)	Cell Cycle	Cell Cycle, Mitotic	Recruitment of mitotic centrosome proteins and complexes	1 (1)	1	0,0091	79	8	1,52E-03	2,18E-01	1,2	3,7	CLASP1:4+0, TUBB4A:2+1, CEP131:2-0, TUBGCP2:2-0, MZ
6	PW(Reactome)	Cell Cycle	Cell Cycle, Mitotic	Loss of proteins required for interphase microtubule organization from the centrosome	1 (1)	1	0,008	69	6	1,17E-02	2,94E-01	1,2	3,7	CLASP1:4+0, TUBB4A:2+1, CEP131:2-0, OFD1:2+0, SSNA1:
7	PW(Reactome)	Cell Cycle	Cell Cycle, Mitotic	Loss of Nlp from mitotic centrosomes	1 (1)	1	0,008	69	6	1,17E-02	2,94E-01	1,2	3,7	CLASP1:4+0, TUBB4A:2+1, CEP131:2-0, OFD1:2+0, SSNA1:
5	PW(Reactome)	Cell Cycle	Cell Cycle, Mitotic	AURKA Activation by TPX2	1 (1)	1	0,0083	72	6	1,41E-02	3,27E-01	1,2	3,7	CLASP1:4+0, TUBB4A:2+1, CEP131:2-0, OFD1:2+0, SSNA1:
4	PW(Reactome)	Cell Cycle	Cell Cycle, Mitotic	Mitotic Prometaphase	1 (1)	1	0,0202	175	10	2,15E-02	3,45E-01	1,2	3,7	CLASP1:4+0, RANGAP1:3-0, TUBB4A:2+1, CEP131:2-0, TUI
5	PW(Reactome)	Cell Cycle	Cell Cycle, Mitotic	Recruitment of NuMA to mitotic centrosomes	1 (1)	1	0,0093	80	8	1,64E-03	2,18E-01	1,2	3,7	CLASP1:4+0, TUBB4A:2+1, CEP131:2-0, TUBGCP2:2-0, MZ
4	PW(Reactome)	Cell Cycle	Chromosome Mainte	Extension of Telomeres	1 (1)	1	0,0035	30	3	4,84E-02	3,51E-01	0,4	3,1	POLD1:3-0, RUVBL2:2-0, WRAP53:2-0
2	PW(Reactome)	Circadian Clock	BMAL1:CLOCK,NPAS2	BMAL1:CLOCK,NPAS2 activates circadian gene expression	1 (1)	1	0,0031	27	3	3,74E-02	3,51E-01	0,3	3,5	ARNTL:4+0, NCOA6:2+0, NCOA1:2+0
6	PW(Reactome)	Gene expression (Transc	RNA Polymerase II Tra	Regulation of TP53 Expression and Degradation	1 (1)	1	0,0034	29	3	4,45E-02	3,51E-01	0,9	4,0	PRDM1:4+1, RFFL:3+0, UBB:2+1
4	PW(Reactome)	Immune System	Cytokine Signaling in I	Interleukin-17 signaling	1 (1)	1	0,0078	67	7	2,49E-03	2,68E-01	0,9	3,1	MEF2C:3-0, JUN:3-1, MAP2K3:3+1, IRAK1:2-0, UBB:2+1, T
5	PW(Reactome)	Immune System	Cytokine Signaling in I	MAP kinase activation	10 (2)	1	0,0068	59	7	1,22E-03	2,18E-01	0,9	3,1	MEF2C:3-0, JUN:3-1, MAP2K3:3+1, IRAK1:2-0, UBB:2+1, T
4	PW(Reactome)	Immune System	Cytokine Signaling in I	Interferon gamma signaling	1 (1)	1	0,0088	76	7	4,92E-03	2,80E-01	2,5	3,5	FCGR1B:4+1, IRF8:3-0, IRF9:3+1, FCGR1A:3+1, HLA-DPA1:
2	PW(Reactome)	Immune System	Adaptive Immune Syst	Adaptive Immune System	1 (1)	1	0,0694	600	25	2,07E-02	3,45E-01	2,5	4,2	LCP2:4+0, FCGR1B:4+1, ACTR1B:3-0, HLA-DOA:3-1, CLTA:
4	PW(Reactome)	Immune System	Adaptive Immune Syst	Generation of second messenger molecules	1 (1)	1	0,0045	39	4	2,20E-02	3,45E-01	2,5	4,2	LCP2:4+0, HLA-DPA1:3-1, HLA-DQB2:3-3, HLA-DRA:2-0
3	PW(Reactome)	Immune System	Adaptive Immune Syst	MHC class II antigen presentation	1 (1)	1	0,0109	94	9	1,15E-03	2,18E-01	2,5	3,3	ACTR1B:3-0, HLA-DOA:3-1, CLTA:3-0, HLA-DPA1:3-1, HLA-
2	PW(Reactome)	Immune System	Innate Immune Syster	Innate Immune System	1 (1)	1	0,1155	998	38	1,83E-02	3,45E-01	1,4	4,2	LCP2:4+0, C1QB:4+1, PFKL:4-0, PCBP2:3+0, ACTR1B:3-0, N
3	PW(Reactome)	Immune System	Innate Immune Syster	Toll-like Receptor Cascades	1 (1)	1	0,0163	141	8	3,88E-02	3,51E-01	0,9	3,1	MEF2C:3-0, JUN:3-1, MAP2K3:3+1, IRAK1:2-0, UBB:2+1, T
4	PW(Reactome)	Immune System	Innate Immune Syster	Toll Like Receptor 4 (TLR4) Cascade	1 (1)	1	0,0134	116	8	1,42E-02	3,27E-01	0,9	3,1	MEF2C:3-0, JUN:3-1, MAP2K3:3+1, IRAK1:2-0, UBB:2+1, T
5	PW(Reactome)	Immune System	Innate Immune Syster	MyD88:MAL(TIRAP) cascade initiated on plasma membrane	3 (1)	1	0,0103	89	7	1,11E-02	2,94E-01	0,9	3,1	MEF2C:3-0, JUN:3-1, MAP2K3:3+1, IRAK1:2-0, UBB:2+1, T
6	PW(Reactome)	Immune System	Innate Immune Syster	MAP kinase activation	10 (2)	1	0,0068	59	7	1,22E-03	2,18E-01	0,9	3,1	MEF2C:3-
5	PW(Reactome)	Immune System	Innate Immune Syster	MyD88-independent TLR4 cascade	1 (1)	1	0,0108	93	8	4,07E-03	2,68E-01	0,9	3,1	MEF2C:3-0, JUN:3-1, MAP2K3:3+1, IRAK1:2-0, UBB:2+1, T
6	PW(Reactome)	Immune System	Innate Immune Syster	TRIF(TICAM1)-mediated TLR4 signaling	1 (1)	1	0,0108	93	8	4,07E-03	2,68E-01	0,9	3,1	MEF2C:3-0, JUN:3-1, MAP2K3:3+1, IRAK1:2-0, UBB:2+1, T
4	PW(Reactome)	Immune System	Innate Immune Syster	Toll Like Receptor 9 (TLR9) Cascade	1 (1)	1	0,0104	90	7	1,18E-02	2,94E-01	0,9	3,1	MEF2C:3-0, JUN:3-1, MAP2K3:3+1, IRAK1:2-0, UBB:2+1, T
5	PW(Reactome)	Immune System	Innate Immune Syster	MyD88 dependent cascade initiated on endosome	2 (1)	1	0,0099	86	7	9,35E-03	2,94E-01	0,9	3,1	MEF2C:3-0, JUN:3-1, MAP2K3:3+1, IRAK1:2-0, UBB:2+1, T
6	PW(Reactome)	Immune System	Innate Immune Syster	TRAF6 mediated induction of NFkB and MAP kinases upon TLR7/8 or 9 activation	2 (1)	1	0,0098	85	7	8,81E-03	2,94E-01	0,9	3,1	MEF2C:3-0, JUN:3-1, MAP2K3:3+1, IRAK1:2-0, UBB:2+1, T
4	PW(Reactome)	Immune System	Innate Immune Syster	Toll Like Receptor 10 (TLR10) Cascade	1 (1)	1	0,0091	79	7	6,03E-03	2,84E-01	0,9	3,1	MEF2C:3-0, JUN:3-1, MAP2K3:3+1, IRAK1:2-0, UBB:2+1, T
5	PW(Reactome)	Immune System	Innate Immune Syster	MyD88 cascade initiated on plasma membrane	2 (1)	1	0,0091	79	7	6,03E-03	2,84E-01	0,9	3,1	MEF2C:3-0, JUN:3-1, MAP2K3:3+1, IRAK1:2-0, UBB:2+1, T
4	PW(Reactome)	Immune System	Innate Immune Syster	Toll Like Receptor 3 (TLR3) Cascade	1 (1)	1	0,0103	89	8	3,14E-03	2,68E-01	0,9	3,1	MEF2C:3-0, JUN:3-1, MAP2K3:3+1, IRAK1:2-0, UBB:2+1, T
4	PW(Reactome)	Immune System	Innate Immune Syster	Toll Like Receptor 5 (TLR5) Cascade	1 (1)	1	0,0091	79	7	6,03E-03	2,84E-01	0,9	3,1	MEF2C:3-0, JUN:3-1, MAP2K3:3+1, IRAK1:2-0, UBB:2+1, T
4	PW(Reactome)	Immune System	Innate Immune Syster	Toll Like Receptor 7/8 (TLR7/8) Cascade	1 (1)	1	0,0099	86	7	9,35E-03	2,94E-01	0,9	3,1	MEF2C:3-0, JUN:3-1, MAP2K3:3+1, IRAK1:2-0, UBB:2+1, T
4	PW(Reactome)	Immune System	Innate Immune Syster	Toll Like Receptor 2 (TLR2) Cascade	1 (1)	1	0,0103	89	7	1,11E-02	2,94E-01	0,9	3,1	MEF2C:3-0, JUN:3-1, MAP2K3:3+1, IRAK1:2-0, UBB:2+1, T
5	PW(Reactome)	Immune System	Innate Immune Syster	Toll Like Receptor TLR1:TLR2 Cascade	1 (1)	1	0,0103	89	7	1,11E-02	2,94E-01	0,9	3,1	MEF2C:3-0, JUN:3-1, MAP2K3:3+1, IRAK1:2-0, UBB:2+1, T
5	PW(Reactome)	Immune System	Innate Immune Syster	Toll Like Receptor TLR6:TLR2 Cascade	1 (1)	1	0,0103	89	7	1,11E-02	2,94E-01	0,9	3,1	MEF2C:3-0, JUN:3-1, MAP2K3:3+1, IRAK1:2-0, UBB:2+1, T
5	PW(Reactome)	Immune System	Innate Immune Syster	IRF3-mediated induction of type I IFN	1 (1)	1	0,0015	13	2	4,87E-02	3,51E-01	0,6	3,2	DTX4:3-1, IFI16:2+0
3	PW(Reactome)	Immune System	Innate Immune Syster	Neutrophil degranulation	1 (1)	1	0,0489	423	18	3,88E-02	3,51E-01	0,9	3,7	PFKL:4-0, ACTR1B:3-0, NEU1:3-0, ARHGAP45:3-0, DPP7:3-
2	PW(Reactome)	Metabolism	Cytosolic iron-sulfur c	Cytosolic iron-sulfur cluster assembly	1 (1)	1	0,0015	13	2	4,87E-02	3,51E-01	0,4	3,1	POLD1:3-0, NUBP2:2-0
4	PW(Reactome)	Metabolism of proteins	Post-translational pro	Metalloprotease DUBs	1 (1)	1	0,0023	20	3	1,74E-02	3,45E-01	0,9	3,2	UIMC1:3+0, MYSM1:2+0, UBB:2+1
3	PW(Reactome)	Metabolism of proteins	Translation	Mitochondrial translation	1 (1)	1	0,0108	93	6	4,16E-02	3,51E-01	0,5	3,3	MRPS28:3-1, MRPL1:2-0, TUFM:2-0, MRPL49:2-0, MRPL52
4	PW(Reactome)	Metabolism of proteins	Translation	Mitochondrial translation elongation	1 (1)	1	0,0102	88	6	3,32E-02	3,51E-01	0,5	3,3	MRPS28:3-1, MRPL1:2-0, TUFM:2-0, MRPL49:2-0, MRPL52
3	PW(Reactome)	Metabolism of RNA	Processing of Capped	mRNA Splicing	1 (1)	1	0,0214	185	10	2,98E-02	3,51E-01	0,4	3,3	PCBP2:3+0, FUS:3+0, PRPF19:3-0, GTF2F1:3-0, LSM4:2-0, I
4	PW(Reactome)	Metabolism of RNA	Processing of Capped	mRNA Splicing - Major Pathway	1 (1)	1	0,0205	177	10	2,30E-02	3,45E-01	0,4	3,3	PCBP2:3+0, FUS:3+0, PRPF19:3-0, GTF2F1:3-0, LSM4:2-0, I
2	PW(Reactome)	Mitophagy	Receptor Mediated M	Receptor Mediated Mitophagy	1 (1)	1	0,0013	11	2	3,61E-02	3,51E-01	0,4	3,7	PGAM5:4-0, MAP1LC3B:2+0
3	PW(Reactome)	Organelle biogenesis an	Cilium Assembly	Anchoring of the basal body to the plasma membrane	1 (1)	1	0,0112	97	7	1,70E-02	3,45E-01	1,2	3,7	CLASP1:4+0, TUBB4A:2+1, CEP131:2-0, C2CD3:2+0, OFD1:
2	PW(Reactome)	Signal Transduction	Death Receptor Signa	Death Receptor Signalling	1 (1)	1	0,0147	127	8	2,30E-02	3,45E-01	0,9	3,9	APH1A:4-0, ARHGEF18:3-0, IRAK1:2-0, UBB:2+1, TAB3:2+0
3	PW(Reactome)	Signal Transduction	Death Receptor Signa	p75 NTR receptor-mediated signalling	1 (1)	1	0,0102	88	6	3,32E-02	3,51E-01	0,9	3,9	APH1A:4-0, ARHGEF18:3-0, IRAK1:2-0, UBB:2+1, ARHGEF1
4	PW(Reactome)	Signal Transduction	Death Receptor Signa	Cell death signalling via NRAGE, NRIF and NADE	1 (1)	1	0,0078	67	5	3,62E-02	3,51E-01	0,9	3,9	APH1A:4-0, ARHGEF18:3-0, UBB:2+1, ARHGEF12:2+1, BAD

Module Pathway / GO Term Enrichment filter settings:

- Pathway / Go Term (Database) [column B]: BP(GO)
- P-value From Gene Set [column AU]: < 1e-4
- Maximum abs(logFC) Nodes [column AW]: > 1.3
- Maximum –log10(P) Hitgenes [column AX]: > 3

Table S5.15.

Hierarchy Pathways and GO Terms					Module Pathway / GO Term Enrichment												
Level	Pathway / GO Term (Database)	Level 1 Pathway / GO Term	Level 2 Pathway / GO Term	Enriched (highest level) Pathway / GO Term	Modules	Main module	NumberOf Repeats InTree	Unique Ancestors InTree	RatioOf Proteins InGeneSe	NumberOf Proteins InGeneSet	Proteins From Modul	P-value From GeneS	FDR From GeneS	Maximum abs(logFC) ModNod	Maximum -10log(P) ModNode	[Module: P-value] Nodes:-10log(P)±log(FC)
7	BP(GO)	biological regulation	regulation of negative regulation of negat	Fc-epsilon receptor signaling pathway	1,5,8	8	6 (2)	1	0,0087	115	3	5,34E-05	1,07E-04	1,3	4,2	[1: 1,75e-01] JUN:3-1, [5: 9,17e-02] TAB3:2+0, [8: 5,34e-05
6	BP(GO)	biological regulation	regulation of negative regulation of negat	complement activation	8	8	10 (5)	1	0,0066	87	3	2,33E-05	7,00E-05	1,4	3,9	[8: 2,33e-05] C1QB:4+1, IGHV3-7:2-1, IGKV1-16:2-1
7	BP(GO)	biological regulation	regulation of negative regulation of negat	complement activation, classical pathw	8	8	10 (5)	1	0,0108	143	5	1,81E-08	5,61E-07	1,4	3,9	[8: 1,81e-08] C1QB:4+1, IGLL5:3-1, IGHV3-7:2-1, IGHM:2-1,
4	BP(GO)	biological regulation	regulation of negative regulation of negat	regulation of complement activation	8	8	3 (1)	1	0,008	105	3	4,08E-05	1,07E-04	1,4	3,9	[8: 4,08e-05] C1QB:4+1, IGHV3-7:2-1, IGKV1-16:2-1
7	BP(GO)	biological regulation	regulation of biological quality	acetylcholine catabolic process in syna	20	20	25 (7)	1	0,0002	2	2	2,29E-08	5,74E-07	1,7	3,2	[20: 2,29e-08] COLQ:3+1, ACHE:2+2
6	BP(GO)	cellular process	cellular metabolic process	acetylcholine catabolic process in syna	20	20	25 (7)	1	0,0002	2	2	2,29E-08	5,74E-07	1,7	3,2	[20: 2,29e-08] COLQ:3+1, ACHE:2+2
5	BP(GO)	cellular process	movement of cell or subcellular compone	leukocyte migration	1,8,16	8	3 (3)	1	0,0142	187	4	4,79E-06	2,87E-05	1,3	3,4	[1: 2,69e-01] SLC3A2:2-0, [8: 4,79e-06] VPREB3:3-1, IGHV3-
6	BP(GO)	cellular process	signal transduction	Fc-epsilon receptor signaling pathway	1,5,8	8	6 (2)	1	0,0087	115	3	5,34E-05	1,07E-04	1,3	4,2	[1: 1,75e-01] JUN:3-1, [5: 9,17e-02] TAB3:2+0, [8: 5,34e-05
3	BP(GO)	immune system process	activation of immune response	complement activation	8	8	10 (5)	1	0,0066	87	3	2,33E-05	7,00E-05	1,4	3,9	[8: 2,33e-05] C1QB:4+1, IGHV3-7:2-1, IGKV1-16:2-1
4	BP(GO)	immune system process	activation of immune response	complement activation, classical pathw	8	8	10 (5)	1	0,0108	143	5	1,81E-08	5,61E-07	1,4	3,9	[8: 1,81e-08] C1QB:4+1, IGLL5:3-1, IGHV3-7:2-1, IGHM:2-1,
5	BP(GO)	immune system process	antigen processing and presentation	antigen processing and presentation of	6	6	4 (1)	1	0,0073	97	9	1,11E-16	6,44E-15	2,5	3,3	[6: 1,11e-16] ACTR1B:3-0, HLA-DOA:3-1, CLTA:3-0, HLA-DP.
3	BP(GO)	immune system process	immune effector process	complement activation	8	8	10 (5)	1	0,0066	87	3	2,33E-05	7,00E-05	1,4	3,9	[8: 2,33e-05] C1QB:4+1, IGHV3-7:2-1, IGKV1-16:2-1
4	BP(GO)	immune system process	immune effector process	complement activation, classical pathw	8	8	10 (5)	1	0,0108	143	5	1,81E-08	5,61E-07	1,4	3,9	[8: 1,81e-08] C1QB:4+1, IGLL5:3-1, IGHV3-7:2-1, IGHM:2-1,
2	BP(GO)	immune system process	immune response	immune response	3,6,8,11,1	8	1 (2)	1	0,0285	376	6	4,16E-08	6,24E-07	1,6	4,2	[3: 2,93e-01] CCR10:2-1, [6: 1,01e-07] HLA-DOA:3-1, HLA-D
4	BP(GO)	immune system process	immune response	complement activation	8	8	10 (5)	1	0,0066	87	3	2,33E-05	7,00E-05	1,4	3,9	[8: 2,33e-05] C1QB:4+1, IGHV3-7:2-1, IGKV1-16:2-1
5	BP(GO)	immune system process	immune response	complement activation, classical pathw	8	8	10 (5)	1	0,0108	143	5	1,81E-08	5,61E-07	1,4	3,9	[8: 1,81e-08] C1QB:4+1, IGLL5:3-1, IGHV3-7:2-1, IGHM:2-1,
2	BP(GO)	immune system process	leukocyte migration	leukocyte migration	1,8,16	8	1 (3)	1	0,0142	187	4	4,79E-06	2,87E-05	1,3	3,4	[1: 2,69e-01] SLC3A2:2-0, [8: 4,79e-06] VPREB3:3-1, IGHV3-
3	BP(GO)	immune system process	production of molecular mediator of imm	immunoglobulin production	8	8	1 (1)	1	0,0039	51	3	4,76E-06	2,87E-05	1,6	3,4	[8: 4,76e-06] VPREB3:3-1, IGKV1-8:2-2, IGKV1-16:2-1
4	BP(GO)	locomotion	cell motility	leukocyte migration	1,8,16	8	3 (3)	1	0,0142	187	4	4,79E-06	2,87E-05	1,3	3,4	[1: 2,69e-01] SLC3A2:2-0, [8: 4,79e-06] VPREB3:3-1, IGHV3-
6	BP(GO)	metabolic process	catabolic process	acetylcholine catabolic process in syna	20	20	25 (7)	1	0,0002	2	2	2,29E-08	5,74E-07	1,7	3,2	[20: 2,29e-08] COLQ:3+1, ACHE:2+2
6	BP(GO)	metabolic process	cellular metabolic process	acetylcholine catabolic process in syna	20	20	25 (7)	1	0,0002	2	2	2,29E-08	5,74E-07	1,7	3,2	[20: 2,29e-08] COLQ:3+1, ACHE:2+2
5	BP(GO)	metabolic process	hormone metabolic process	acetylcholine catabolic process in syna	20	20	25 (7)	1	0,0002	2	2	2,29E-08	5,74E-07	1,7	3,2	[20: 2,29e-08] COLQ:3+1, ACHE:2+2
5	BP(GO)	metabolic process	nitrogen compound metabolic process	acetylcholine catabolic process in syna	20	20	25 (7)	1	0,0002	2	2	2,29E-08	5,74E-07	1,7	3,2	[20: 2,29e-08] COLQ:3+1, ACHE:2+2
6	BP(GO)	metabolic process	organic substance metabolic process	acetylcholine catabolic process in syna	20	20	25 (7)	1	0,0002	2	2	2,29E-08	5,74E-07	1,7	3,2	[20: 2,29e-08] COLQ:3+1, ACHE:2+2
2	BP(GO)	response to stimulus	immune response	immune response	3,6,8,11,1	8	1 (2)	1	0,0285	376	6	4,16E-08	6,24E-07	1,6	4,2	[3: 2,93e-01] CCR10:2-1, [6: 1,01e-07] HLA-DOA:3-1, HLA-D
4	BP(GO)	response to stimulus	immune response	complement activation	8	8	10 (5)	1	0,0066	87	3	2,33E-05	7,00E-05	1,4	3,9	[8: 2,33e-05] C1QB:4+1, IGHV3-7:2-1, IGKV1-16:2-1
5	BP(GO)	response to stimulus	immune response	complement activation, classical pathw	8	8	10 (5)	1	0,0108	143	5	1,81E-08	5,61E-07	1,4	3,9	[8: 1,81e-08] C1QB:4+1, IGLL5:3-1, IGHV3-7:2-1, IGHM:2-1,

A.6. R-studio scripts

Script S6.1.

For both hierarchical clustering heatmap of top 20 differentially expressed genes in DM1 OPTIMISTIC and DMBDI G2vsG1.

```
# Heatmap.2 is not available for this R version, but please make use of gplots which includes Heatmap.2
### A) Installing and loading required packages -> install packages anyway, which has to do
###    with the dependencies = TRUE
# require("gplots"))
install.packages("gplots", dependencies = TRUE)
library(gplots)
# require("RColorBrewer"))
install.packages("RColorBrewer", dependencies = TRUE)
library(RColorBrewer)
library(tidyverse)                          # data manipulation
library(cluster)                            # clustering algorithms
library(factoextra)                         # clustering algorithms & visualization
### B) Reading in data and transform it into matrix format
data <- read.csv("/Users/…………../Desktop/…….csv", sep=",")
rnames <- data[,1]                                  # assign labels in column 1 to "rnames"
mat_data <- data.matrix(data[,2:ncol(data)])        # transform column 2-20 into a matrix
rownames(mat_data) <- rnames                        # assign row names
Ydata = t(mat_data)
Ymat_data = scale(Ydata)
Xmat_data = t(Ymat_data)
## Row- and column-wise clustering
hr <- hclust(as.dist(1-cor(Ymat_data, method="pearson")), method="complete")
hc <- hclust(as.dist(1-cor(Xmat_data, method="pearson")), method="complete")
mycl <- cutree(hr, h=max(hr$height)/1.008); mycolhc <- rainbow(length(unique(mycl)), start=0.1, end=0.9);
mycolhc <- mycolhc[as.vector(mycl)]
cluster <- as.matrix(mycl)
output<- cbind(Xmat_data,cluster)
### C) Customizing and plotting the heatmap
# creates a own color palette from red to blue
my_palette <- colorRampPalette(c("blue", "white", "red"))(256)
heatmap.2(Xmat_data,
      main = "",                      # heatmap title
      notecol="black",                # change font color of cell labels to black
      density.info="none",            # turns off density plot inside color legend
      xlab="Subjects with DM1",
      ylab="Differentially expressed genes",
      labCol = "",
      labRow = row.names(Xmat_data),
      trace="none",                   # turns off trace lines inside the heatmap
      margins =c(8,18),               # widens margins around plot
      col=my_palette,                 # use on color palette defined earlier
      key.par=list(mar=c(6,2,4,9)),
      key.xlab = "Column Z-score",
      cexRow = 1.4,
      cexCol = 1.2,
      Rowv=as.dendrogram(hr), Colv=as.dendrogram(hc),
      dendrogram=c("column"),                     # only draw a column dendrogram
      ColSideColors = rep(c("gray", "black"), each = 10),   # grouping row-variables into different
                                           # Group 1 samples 1-10: gray
                                           # Group 2 samples 11-20: black
      title = "Groups")               # heatmap title
                                      # adding a color legend for the groups
      # par(lend = 1)                 # square line ends  for the color legend
    legend("topright",                # location of the legend on the heatmap plot
    legend = c("Group 1: CTG repeat lengths ≤400; MIRS 1-2", "Group 2: CTG repeat lengths ≤400; MIRS 3-    5"),
                                       # category labels
    col = c("gray", "black"),          # color key
    lty = 1,                           # line style
    lwd = 20                           # line width
)
```

S6.2. script

P-value distribution plot OPTIMISTIC G2vsG1
OPTIM_G2vsG1V1 <- read.table("/Users/.../Desktop/OPTIMISTIC_G2vsG1V1.txt", sep = "\t", header = T, row.names = 1)
hist(OPTIM_G2vsG1V1$P.Value, xlab= "p-values", ylim=c(0, 2500), cex.axis = 1.3, cex.lab=1.5, col = "blue1")

S6.3. script
P-value distribution plot OPTIMISTIC G2vsG1
OPTIM_G3vsG2V1<-read.table("/Users/…/Desktop/OPTIMISTIC_G3vsG2V1.txt", sep = "\t", header = T, row.names = 1)
hist(OPTIM_G3vsG2V1$P.Value, xlab= "p-values", ylim=c(0, 2500), cex.axis = 1.3, cex.lab=1.5, col = "blue1")

S6.4. script
P-value distribution plot DMBDI G2vsG1
DMBDI_G2vsG1V1 <- read.table("/…/Desktop/DMBDI_G2vsG1V1.txt", sep = "\t", header = T, row.names = 1)
hist(DMBDI_G2vsG1V1$P.Value, xlab= "p-values", ylim=c(0, 2500), cex.axis = 1.3, cex.lab=1.5, col = "blue1")

S6.5. script
Hierarchical clustering heatmap of all 683 differentially expressed genes in OPTIMISTIC DM1 G2vsG1

```
# Heatmap.2 is not available for this R version, use gplots which includes Heatmap.2
### A) Installing and loading required packages
### with the dependencies = TRUE
# require("gplots"))
install.packages("gplots", dependencies = TRUE)
library(gplots)
# require("RColorBrewer"))
install.packages("RColorBrewer", dependencies = TRUE)
library(RColorBrewer)
library(tidyverse)                    # data manipulation
library(cluster)                      # clustering algorithms
library(factoextra)                   # clustering algorithms & visualization

### B) Reading in data and transform it into matrix format

data <- read.csv("/Users/.../Desktop/...csv", sep=",")
rnames <- data[,1]                                  # assign labels in column 1 to "rnames"
mat_data <- data.matrix(data[,2:ncol(data)])        # transform column 2-20 into a matrix
rownames(mat_data) <- rnames                        # assign row names
Ydata = t(mat_data)
Ymat_data = scale(Ydata)
Xmat_data = t(Ymat_data)
## Row- and column-wise clustering
hr <- hclust(as.dist(1-cor(Ymat_data, method="pearson")), method="complete")
hc <- hclust(as.dist(1-cor(Xmat_data, method="pearson")), method="complete")
mycl <- cutree(hr, h=max(hr$height)/1.008); mycolhc <- rainbow(length(unique(mycl)), start=0.1, end=0.9); mycolhc <- mycolhc[as.vector(mycl)]
cluster <- as.matrix(mycl)
output<- cbind(Xmat_data,cluster)

### C) Customizing and plotting the heatmap
# creates own color palette from red to blue
my_palette <- colorRampPalette(c("red", "white", "blue"))(256)
heatmap.2(Xmat_data,
        main = "",                    # heatmap title
        notecol="black",              # change font color of cell labels to black
        density.info="none",          # turns off density plot inside color legend
        xlab="Subjects with DM1",
        ylab="Differentially expressed genes",
        labCol = "",
        labRow = "",
        trace="none",                 # turns off trace lines inside the heatmap
        margins =c(8,10),             # widens margins around plot
        col=my_palette,               # use on color palette defined earlier
        key.par=list(mar=c(6,2,4,9)),
        key.xlab = "Column Z-score",
        cexRow = 1.4,
        cexCol = 1.2,
        Rowv=as.dendrogram(hr), Colv=as.dendrogram(hc),
        dendrogram=c("column"),                       # only draw a column dendrogram
```

```
        ColSideColors = rep(c("gray", "black"), each = 10),     # grouping row-variables into different
                                                                  # Group 1 samples 1-10: gray
                                                                  # Group 2 samples 11-20: black
        title = "Groups")                                         # heatmap title
# adding a color legend for the groups
# par(lend = 1)                          # square line ends  for the color legend
legend("topright",                       # location of the legend on the heatmap plot
legend = c("Group 1: CTG repeat lengths ≤400; MIRS 1-2", "Group 2: CTG repeat lengths ≤400; MIRS 3-5"),
                                         # category labels
col = c("gray", "black"),                # color key
lty = 1,                                 # line style
lwd = 20                                 # line width
)
```

S7. Splice variant analysis

FASTQ files were trimmed and quality checked by Trim Galore! (v0.4.4_dev), which is a wrapper tool around Cutadapt (v1.18) [3] and FastQC (v0.11.5). STAR (v2.6.0a) [4], a splice aware aligner, was used for aligning the trimmed FASTQ files to the reference genome. Ensembl human genome 38 release 95 (GRCh38.95) was used as reference annotation. STAR was run with the following non-default parameters: readFilesCommand gunzip -c, outSamtype BAM Unsorted, outSJfilterReads Unique (only for DM1 data). Two tools were used to identify splicing events discriminating the two groups: LeafCutter (branch: "newstanlayout"; [5], which focuses on the detection of differences in intron excision, and DEXseq (v1.28.3; [5], which focuses on differences in exonic coverage (corrected for potential differences in total gene expression). Both LeafCutter and DEXSeq used bam files as input. Every cluster in LeafCutter had to be supported by at least fifty reads, allowing introns with a maximum length of 500kb. Differential intron excision was determined, requiring at least two samples per group to have a minimal coverage of thirty reads per intron cluster, ignoring introns used by fewer than two samples. Output of the differential intron excision was filtered on an adjusted P-value of lower than 0.05 and status being 'Success'. The visualization of the LeafCutter results was done using an interactive browser-based application, built on the R Shiny framework, included with LeafCutter. The Ensembl reference annotation GRCh38.95 was used for the visualization and the FDR filter was set to 0.05. The splicing analysis with LeafCutter was controlled for a gender covariate in the DM1 data, by including an extra column in the *group/design* file.
DEXSeq is dependent on a reference annotation to provide information about exons. A modified version of the Ensembl GRCh38.95 annotation was used for this to prevent doing analysis on very small exon bins. Partly overlapping exons in the annotation were combined into merged exons (Figure S2.7.). Using python3 the Ensembl annotation was loaded, extracting lines containing annotation for genes and exons. All unique exons, having unique start and end positions, belonging to a gene were collected and checked for overlapping exons. If two exons were to overlap, according to the formula

$$\max(i_{start}, j_{start}) \leq \min(i_{start}, j_{start}) \quad (1)$$

where *i* is the first exon and *j* the second, the two exons were combined into a new merged exon by taking the lowest start and the highest stop as the new values, removing the old exons. Once the line annotating for the next gene was encountered, the process was repeated until the whole file was done. Next the new annotation file was created based on the merged exons.
As we used bam files as input for the splicing analysis, the file flag was set to bam when counting reads; *-f bam*. The resulting data frame from the DEXSeq splicing analysis was filtered on an adjusted P-value lower than 0.05 **and an absolute log2 fold change higher than 1.0. Plots were made of the remaining exon bins using the provided plot function, with** the FDR set to 0.05. DEXSeq was controlled for a gender co-variate in the DM1 data by including an extra column to the design file and by providing a full model (2) and a reduced model (3), including an interaction term of gender with exon.

fullModel = ~ sample + exon + **gender:exon** + condition:exon (2)

reducedModel = ~ sample + exon + **gender:exon** (3)

S8.1. Supplementary list of all 683 differentially expressed genes in the OPTIMISTIC G2 vs G1 dataset.
Table S8.1. See excel-file Reference [7].

S8.2. Supplementary list of all differentially expressed genes in the DMBDI G2 vs G1 dataset to create heatmap Figure 2 from the main text, see excel-file Reference [7].

S9. OPTIMISTIC Master regulators
Table S9.1. See excel-file Reference [7].

S10. DMBDI Master regulators
Table S10.1. See excel-file Reference [7].

References

1. Okkersen, K.; Jimenez-Moreno, C.; Wenninger, S.; Daidj, F.; Glennon, J.; Cumming, S.; Littleford, R.; Monckton, D.G.; Lochmuller, H.; Catt, M.; et al. Cognitive behavioural therapy with optional graded exercise therapy in patients with severe fatigue with myotonic dystrophy type 1: a multicentre, single-blind, randomised trial. *The Lancet. Neurology* **2018**, *17*, 671-680, doi:10.1016/s1474-4422(18)30203-5.
2. Krämer, A.; Green, J.; Pollard, J., Jr.; Tugendreich, S. Causal analysis approaches in Ingenuity Pathway Analysis. *Bioinformatics (Oxford, England)* **2014**, *30*, 523-530, doi:10.1093/bioinformatics/btt703.
3. Martin, M. Cutadapt removes adapter sequences from high-throughput sequencing reads. *2011* **2011**, *17*, 3, doi:10.14806/ej.17.1.200.
4. Dobin, A.; Davis, C.A.; Schlesinger, F.; Drenkow, J.; Zaleski, C.; Jha, S.; Batut, P.; Chaisson, M.; Gingeras, T.R. STAR: ultrafast universal RNA-seq aligner. *Bioinformatics (Oxford, England)* **2013**, *29*, 15-21, doi:10.1093/bioinformatics/bts635.
5. Li, Y.I.; Knowles, D.A.; Humphrey, J.; Barbeira, A.N.; Dickinson, S.P.; Im, H.K.; Pritchard, J.K. Annotation-free quantification of RNA splicing using LeafCutter. *Nat Genet* **2018**, *50*, 151-158, doi:10.1038/s41588-017-0004-9.
6. DM1 enrichment results.xlsm
7. Supplementary_S. Nieuwenhuis.xlsx
